# Direct reciprocity and model-predictive rationality explain network reciprocity over social ties

**DOI:** 10.1038/s41598-019-41547-w

**Published:** 2019-04-01

**Authors:** Fabio Dercole, Fabio Della Rossa, Carlo Piccardi

**Affiliations:** Department of Electronics, Information, and Bioengineering, Politecnico di Milano, Piazza Leonardo da Vinci 32, I-20133 Milano, Italy

## Abstract

Since M. A. Nowak & R. May’s (1992) influential paper, limiting each agent’s interactions to a few neighbors in a network of contacts has been proposed as the simplest mechanism to support the evolution of cooperation in biological and socio-economic systems. The network allows cooperative agents to self-assort into clusters, within which they reciprocate cooperation. This (induced) network reciprocity has been observed in several theoreticalmodels and shown to predict the fixation of cooperation under a simple rule: the benefit produced by an act of cooperation must outweigh the cost of cooperating with all neighbors. However, the experimental evidence among humans is controversial: though the rule seems to be confirmed, the underlying modeling assumptions are not. Specifically, models assume that agents update their strategies by imitating better performing neighbors, even though imitation lacks rationality when interactions are far from all-to-all. Indeed, imitation did not emerge in experiments. What did emerge is that humans are conditioned by their own mood and that, when in a cooperative mood, they reciprocate cooperation. To help resolve the controversy, we design a model in which we rationally confront the two main behaviors emerging from experiments—reciprocal cooperation and unconditional defection—in a networked prisoner’s dilemma. Rationality is introduced by means of a predictive rule for strategy update and is bounded by the assumed model society. We show that both reciprocity and a multi-step predictive horizon are necessary to stabilize cooperation, and sufficient for its fixation, provided the game benefit-to-cost ratio is larger than a measure of network connectivity. We hence rediscover the rule of network reciprocity, underpinned however by a different evolutionary mechanism.

## Introduction

Cooperation among self-interested agents is a longstanding and still debated puzzle in biology and social sciences, with countless contributions since R. Axelrod, W. D. Hamilton, and R. L. Trivers’ seminal works^1,2^ (see ref.^3^ for a recent review on human cooperation); the topic has also received attention in several fields of engineering^4^.

The standard modeling framework is *evolutionary game theory* (EGT)^5–7^, in which a game describes the interaction among pairs (or a larger groups^8^) of self-interested agents in a population, a given set of behavioral strategies is confronted, and an evolutionary process links the obtained payoffs to reproduction and death in biology or to strategy update in socio-economic systems. The paradigmatic game used to study the evolution of cooperation is the *prisoner’s dilemma* (PD), the two-player-two-option interaction in which a cooperator (option C) provides a benefit *b* to the opponent at a cost $c < b$ to herself, whereas a defector (option D) provides no benefit at no cost. The *benefit-to-cost* ratio, or PD *return* $r=b/c$, is often used to parameterize the game, taking $c=1$ as monetary unit (see SI note 1 for other parameterizations). Compared to other social dilemmas, the PD is considered the worst-case for the evolution of cooperation^5–7^ (SI note 2).

The standard way to test whether a cooperative strategy (strategy C) has any chance to evolve, is to confront it with the benchmark strategy ‘unconditional defection’ (strategy D; agents who always defect) in playing the PD under one or a few evolutionary processes. The three different issues to be discussed are: the *invasion* of the strategy, i.e., the spreading of cooperators (C-agents, or C’s) in a population dominated by defectors (D-agents, or D’s); its *persistence*, i.e., the long-term stabilization, fluctuating or not, of C’s; and its *fixation*, i.e., the convergence to the state all-C. For example, it is well known that when the PD is played in large and well-mixed— *unstructured*—populations, there is no hope for the strategy ‘unconditional cooperation’ (agents who always cooperate) against the benchmark D-strategy. Defecting gives the largest payoff regardless of what the others are doing; thus, without any specific incentive to cooperation, C’s cannot invade under any reasonable evolutionary process and disappear if initially present in the population.

Traditional incentivizing mechanisms^9^ either make cooperation conditional—such as reciprocal altruism^1,2^ (also known as *direct reciprocity*), the establishment of reputations^10^ (also known as indirect reciprocity), mechanisms of kin^11^ or group^12^ selection (or other forms of assortment^13,14^), and the consideration of social^15,16^ and moral^17^ preferences—or change the rules of the game, as by introducing optional participation^18,19^ and punishment of antisocial behaviors^20,21^. All these mechanisms add degrees of strategical complexity, either in terms of players’ cognitive abilities and/or information flows, or due to extra options in the underlying game.

**Figure 1:**
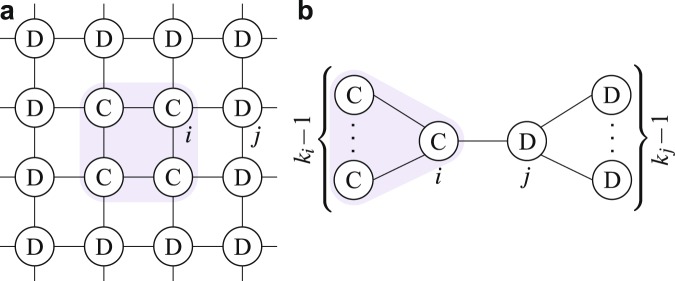
Network reciprocity in regular and non-regular networks. (**a**) A cluster of 4 unconditional C’s surrounded by unconditional D’s in a square lattice with periodic boundary conditions. The payoff (per game round, obtained by each agent by playing a PD with all neighbors and summing up outcomes) of C’s is ${\pi }_{i}=2r-4$; that of the D’s at the boundary of the cluster is ${\pi }_{j}=r$. C’s do better than D’s (${\pi }_{i} > {\pi }_{j}$) if $r > 4$, so that, under imitation update, C’s remain such and boundary D’s change to C (e.g., *j* copying *i*), reducing however their payoff to *r* − 4. (**b**) A cluster of *k*_*i*_ C’s protected from *k*_*j*_ D’s (${k}_{i},{k}_{j} > 2$; *k*_*i*_ and *k*_*j*_ are the degrees of nodes *i* and *j*). The payoff of the C-agent *i* is ${\pi }_{i}=r({k}_{i}-1)-{k}_{i}$; that of the D-agent *j* is ${\pi }_{j}=r$; ${\pi }_{i} > {\pi }_{j}$ if $r > 1+2/({k}_{i}-2)$, so that, under imitation update, *i* remains C and *j* drastically reduces her payoff when copying *i*.

Starting with M. A. Nowak and coauthors’ influential papers^22–24^, the fact that interactions in real populations are *structured* according to a network of physical or social contacts has been proposed as the simplest mechanism—requiring no strategical complexity—to explain cooperation. It has been named *network reciprocity* because theoretical models show that if the network is far from all-to-all (technically *sparse*, i.e. with average *degree* $\langle k\rangle$—the average number of neighbors—significantly lower than the number *N* of nodes), it allows C-agents to self-assort into clusters within which they cooperate. That is, the network induces mutual cooperation between C’s, even with no (direct) reciprocity built-in in their strategy (Fig. 1). More specifically, the original model of network reciprocity works as follows^24^. In large regular networks (where each node has *k* neighbors), driven by an imitative evolutionary process (agents imitate better performing neighbors, see SI note 3), and in the limit of weak selection (the game payoff marginally impacts the individual performance, see SI note 4), unconditional C’s can invade unconditional D’s and fixate under a simple condition: the PD return *r* must exceed the connectivity *k*. This condition has been generalized to non-regular networks^25,26^, essentially requiring *r* to exceed the average degree $\langle k\rangle$.

Network reciprocity has been confirmed in several other models^27–43^ (with a few exceptions, see, e.g., ref.^44^). In contrast, the experimental evidence among humans is controversial (see ref.^3^ for a review). Some authors^45–50^ questioned network reciprocity as a mechanism supporting human cooperation, because experiments on several networks (including a few impressively large regular and non-regular sparse networks^45,47^) gave low levels of cooperation, similar to those observed with all-to-all interaction. However, most of these experiments failed to satisfy the condition $r > \langle k\rangle$, and more recent experiments satisfying the condition showed higher levels of cooperation, in accordance with the theoretical prediction^51,52^.

There is nonetheless another argument fueling the apparent mismatch between theory and practice. While all models confirming network reciprocity assume an imitative process of strategy update, the in-depth analysis of some of the experiments showed that human subjects did not take decisions by comparing with neighbors’ payoffs^46,50^. Imitation indeed lacks rationality when interaction patterns are limited and possibly heterogeneous: Why should we copy a better performing neighbor whose neighborhood might be considerably different—in size as well as in composition—from ours? Especially in heterogeneous networks (networks encompassing nodes with very low and high degree), imitation may turn counterproductive (see Fig. 1, where agent *j* reduces her payoff by copying *i*). Not surprisingly, by changing the rule for strategy update from imitation to ‘best response’—the simplest, though myopic, rational rule of doing what is best for ourself in the next game round—network reciprocity no longer works^53^, as unconditional defection dominates the PD in any network.

In conclusion, the rule $r > \langle k\rangle$ of network reciprocity apparently works, but the underlying theoretical justification seems not. To help resolving the controversy, we design a model that shows network reciprocity, while being more adherent to the common rationale and to the available experiments than previous models based on imitation update. Before describing our model, let us be more precise on what we mean by the fact that an EGT setup, whether theoretical or experimental, shows network reciprocity. We think it is worth to conceptually separate the network from the, possibly induced, reciprocity. The network is the mechanism limiting agents’ interactions. Whether this mechanism does enhance mutual cooperation—reciprocity—or not, with respect to the case of all-to-all interaction, evidently depends on the setup details. For example, the answer is ‘yes’ for the PD played, under $r > \langle k\rangle$, by unconditional C’s and D’s in models based on imitation; ‘no’ if the update rule is changed to best response. In other words, we say that the EGT setup shows network reciprocity if a condition qualitatively similar to $r > \langle k\rangle$ holds true, i.e., if cooperation invades, persists, or even fixates in the population, provided the game return is sufficiently large relative to a measure of network connectivity. In this line of thinking, network reciprocity is evidently an outcome of the EGT setup, not a mechanism built-in in the setup itself.

We do not aim at fitting any of the available experiments. Actually, what we take from them is the pairwise PD interaction, mostly used, and three general emerging facts. The first is that humans are conditioned by their current ‘mood’ to be cooperative or defective; the second is that, while in the C-mood, they tend to reciprocate cooperation; the third is that they defect rather unconditionally while in the D-mood. This evidence justifies our assumption of two strategies, a C-strategy implementing a form of direct reciprocity, and the benchmark D-strategy. We implement direct reciprocity by giving C-agents a temporary exit option^18,54^ toward neighboring defectors, so to reduce exploitation risk. As in the famous tit-for-tat strategy, traditionally used to model direct reciprocity^2^, C’s cooperate with neighbors known to cooperate. However, they do not retaliate for defection; rather, they abstain from playing for a few rounds; eventually, they forgive defection and go back playing to seek cooperation, i.e., they poll previous exploiters for a change of mood. Abstention has no cost and gives no benefit to both opponents of the interaction, and we assume it can be distinguished from defection. This is because we imagine interactions taking place in a physical or virtual space in which one notes whether the opponent shows up or not, though identities might not be disclosed. This means that, by abstaining, an agent communicates her C-mood to the opponent, provided the latter shows up. At the same time, abstention provides no information on the opponent’s mood.

The rule for strategy update is where we embed rationality in our model. Following the standard EGT tenet of selfishness, we extend the best response rule to the prediction of future incomes over a multi-step horizon of $h\ge 2$ future interactions. The prediction is based on our model, that sets the rules of the society and that is assumed to be common knowledge to all agents. The only further available information directly comes from the pairwise interactions. No access is given to neighbors’ payoffs and connectivity. Each agent revises her current strategy from time to time, independently of others, in accordance with a rate *δ* assumed uniform across the population. Technically, *δ* is the probability of strategy update after each game round; 1/*δ* measures the agents’ ‘inertia’ to change (the average number of rounds between two consecutive updates by the same agent). When revising the strategy, an agent computes the cumulated income expected from using the C and D strategies over the predictive horizon, and selects the best one up to the next update. Because of the limited information, the prediction cannot account for changes in the neighbors’ strategies. This limits the horizon to a few rounds under a relatively slow strategy update (small *δ*); because of the short horizon, no discount of future incomes is adopted.

Based on the update rate *δ*, we set the measure for direct reciprocity. We say that ‘normally’ reciprocating C-agents are those who, at each round of an abstention period, decide whether to go back playing in accordance with the expectation that the neighboring defector has revised her strategy. ‘Super/sub’-reciprocating C’s abstain for longer/shorter periods, $\langle a\rangle$ on average. They do so by intentionally under/over-biasing their neighbors’ update rate to ${\delta }_{d}=(1-d)\delta$, where −100*d*% is the negative/positive bias. Parameter *d* modulates the strength of direct reciprocity, from no reciprocity at $d={d}_{{\rm{\min }}}=-\,(1/\delta -1)$, yielding ${\delta }_{d}=1$ and the unconditional C strategy, to extreme reciprocity at $d={d}_{{\rm{\max }}}=1$, yielding ${\delta }_{d}=0$ and C’s who definitely break connections with defecting neighbors; $d=0$ is the unbiased case of normal reciprocity. Correspondingly, we say that direct reciprocity is super/sub-normal if *d* is positive/negative.

We close this introduction by anticipating that this particular form of direct reciprocity is not crucial for our results. We use it because we consider abstention, whether possible, more connatural to the cooperative mood than retaliation. This issue is addressed in the discussion, where we further comment on the novelty and scope of our model. Overall, the model-predictive rule for strategy update is the new key element, that we use to describe a rational decision making. Further details on the model’s implementation are given in the Methods. The model parameters are summarized in Table 1.

**Table 1:** Model parameters (first part) and related quantities (second part).

Parameter	Description	Reference values	Other values
*N*	network size	1000	—
*r*	PD return	$[1,6]$	$[20,60]$ in Fig. S2e
*δ*	rate of strategy update	0.05	0.025, 0.1 in Fig. S3 (right)
*h*	predictive horizon	2, 3, 4, 5	—
*d*	direct reciprocity (${d}_{{\rm{\min }}}=-\,(1/\delta -1)$; ${d}_{{\rm{\max }}}=1$)	0 (normal)	1/2 (super), −1 (sub) in Fig. S3 (left)
${\delta }_{d}=(1-d)\delta$	reciprocity-biased update rate	0.05	0.025, 0.1 in Fig. S3 (left)
$\langle a\rangle$	average length of abstention periods	4.56	6.90, 2.91 in Fig. S3

## Results

**Figure 2:**
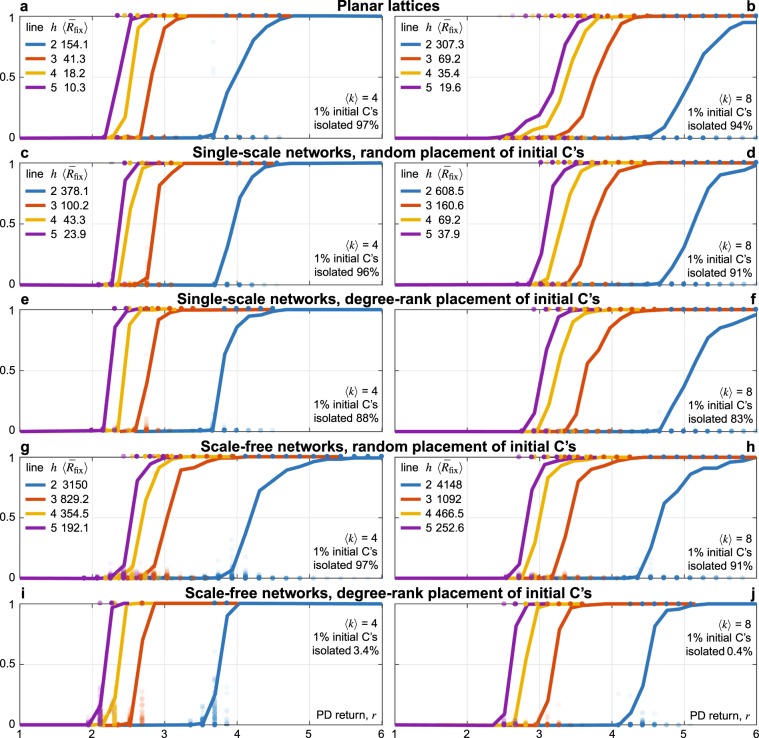
Invasion, persistence, and fixation of cooperation under direct reciprocity and model-predictive rationality. Panels show the level of cooperation reached in 10^4^ game rounds starting from 1% inital C’s on different network structures (left: average degree $\langle k\rangle =4$; right: $\langle k\rangle =8$) as a function of the PD return *r*. Solid lines show the average fraction over 100 random initializations (random placement of the initial C’s in planar lattices; network generation and random placement of the initial C’s for random—single scale and scale free—networks; the average % of isolated initial C’s is reported). Dots show the outcome of single simulations (only for ${r}_{{\rm{inv}}} < r < {r}_{{\rm{fix}}}$, i.e., only if some of the outcomes lie in the open interval (0, 1)); transparency is used to show dots accumulation. Colors code the predictive horizon *h*, from 2 to 5, and the corresponding upper bound ${\bar{R}}_{{\rm{fix}}}$ to the threshold *R*_fix_ is reported. Model parameters: reference values in Table 1. See Sects S11 and S12 for further details on networks and numerical simulations.

Our main result is that cooperation fixates in our model starting from any cluster of at least two C-agents, provided that the PD return *r* is larger than a fixation threshold *R*_fix_ that increases with the maximal degree *k*_max_ in the network. The threshold *R*_fix_ is derived analytically and is very conservative. It represents the worst-case with respect to the position of the initial C’s in the network. In practice, the fixation threshold is more related to the network’s average degree $\langle k\rangle$. This is shown numerically in Fig. 2, in which the actual threshold *r*_fix_ is identified by averaging over many simulations, and shown to increase by doubling the average degree from 4 to 8 in several network structures (planar lattices; single-scale: networks with narrow degree distribution; scale-free: broad degree distribution; see Sect. Numerical results for further details). We hence rediscover the rule of network reciprocity over social ties, explained however by direct reciprocity and model-predictive rationality.

Secondary results of our model, proved analytically and quantitatively confirmed numerically, are listed below.

1. Direct reciprocity ($d > {d}_{{\rm{\min }}}$) is essential for the evolution of cooperation. Moreover, the stronger is reciprocity (larger $d\in ({d}_{{\rm{\min }}},{d}_{{\rm{\max }}})$), the better is for cooperation, in the sense of a reduced fixation threshold.
2. The multi-step prediction ($h\ge 2$) is essential as well, and extending the horizon (larger *h*) helps cooperation.
3. Cooperation can invade, even starting from a single C-agent. With a single C, the fixation granted by the threshold *R*_fix_ occurs with a probability that grows from 1/2 to 1 with the degree of the initial C. This is particularly interesting, as models based on imitation update require a significant initial fraction of C’s to stabilize cooperation (the issue is further addressed in the Discussion).
4. The degree heterogeneity of the network does not help cooperation, unless the initial C’s occupy the network’s hubs (Fig. 2: compare the same networks under the two types of placement—random and degree-rank—of the initial C’s). Both these effects turned out to be rather limited, in agreement with experimental observations^47,48^, while in sharp contrast with the theoretical predictions based on imitation update.

### Analytical results

In this section we present the properties of our model’s dynamics that we proved analytically (some of the proofs are reported in the Supplementary Information). We preliminary consider the case of an infinite predictive horizon. Although lacking sense in our model (because predictions do not account for changes in the neighbors’ strategies, see Introduction), it allows to derive a simple condition for the fixation of cooperation. We prove (in SI Sects S1–S6) that when a C-agent with degree *k* and *k*_C_ C-neighbors (known from past interactions) revises her strategy according to an infinite horizon, she remains C (because she expects a loss in changing to D) if and only if

1 $$r > 1+\frac{k}{{k}_{{\rm{C}}}}\frac{{P}_{{\rm{CD}}}^{\infty }}{1-{P}_{{\rm{CD}}}^{\infty }},\,\,{P}_{{\rm{CD}}}^{\infty }=\frac{1}{2}\frac{\sqrt{4{\delta }_{d}-3{\delta }_{d}^{2}}-{\delta }_{d}}{1-{\delta }_{d}}\simeq \sqrt{{\delta }_{d}}\,{\rm{for}}\,{\rm{small}}\,{\delta }_{d},\,\,{\delta }_{d}=(1-d)\delta \in (0,1).$$

${P}_{{\rm{CD}}}^{\infty }$ is the probability (computed in SI Sect. S2) that a C-agent who remains C forever will get exploited in a far-future interaction by a D-neighbor who remains D forever. It only depends on the reciprocity-biased update rate *δ*_*d*_ and increases from zero to one with *δ*_*d*_ (SI Fig. S8).

Similarly, the D-to-C strategy change (expected gain in changing to C) occurs under the same condition (1). The harshest condition in (1), i.e., the one for $k={k}_{{\rm{\max }}}$ and ${k}_{{\rm{C}}}=1$, hence gives the threshold *R*_fix_ of our main result, in the case of an infinite horizon:

2 $${R}_{{\rm{fix}}}^{\infty }=1+{k}_{{\rm{\max }}}\frac{{P}_{{\rm{CD}}}^{\infty }}{1-{P}_{{\rm{CD}}}^{\infty }}.$$

With a finite horizon of *h* future interactions, the conditions governing strategy update are more complex. In SI Sects S4 and S5, we compute the gains ${\rm{\Delta }}{\pi }_{{\rm{C}}}^{h}$ and ${\rm{\Delta }}{\pi }_{{\rm{D}}}^{h}$ (positive for a gain; negative for a loss) respectively predicted by a C and a D for changing strategy. The resulting expressions are reported in the Methods. Given a C and a D with identical neighborhoods (including ${k}_{{\rm{C}}}\ge 1$ C’s), the *r*-threshold for a C to remain C and that for a D to change to C are different. Typically, the former is lower, because the C-neighbors of a C more likely cooperate in the near future than the C-neighbors of a D.

Not surprisingly, for $h=1$ (best response update) we have ${\rm{\Delta }}{\pi }_{{\rm{C}}}^{1} > 0$ and ${\rm{\Delta }}{\pi }_{{\rm{D}}}^{1} < 0$ independently of the PD return *r* and of the neighborhood’s composition, so that defecting assures the highest payoff. However, for any $h\ge 2$ and provided the agent has a C-neighbor (${k}_{{\rm{C}}}\ge 1$), the expected gains ${\rm{\Delta }}{\pi }_{{\rm{C}}}^{h}$ and ${\rm{\Delta }}{\pi }_{{\rm{D}}}^{h}$ depend linearly on *r*, respectively decreasing and increasing with positive and negative values at $r=1$. This shows the existence of the threshold *R*_fix_, an upper bound of which is provided in SI Sect. S7 by considering the network’s node with maximal degree.

Finally, in SI Sect. S8, we further analyze the role of the predictive horizon. Under a condition on *r* more restrictive than (1), the expected gains ${\rm{\Delta }}{\pi }_{{\rm{C}}}^{h}$ and ${\rm{\Delta }}{\pi }_{{\rm{D}}}^{h}$ depend monotonically on *h*, respectively decreasing and increasing to the negative and positive infinite-horizon limits. For intermediate *r*, ${\rm{\Delta }}{\pi }_{{\rm{C}}}^{h}$ (resp. ${\rm{\Delta }}{\pi }_{{\rm{D}}}^{h}$) first increases (decreases) with *h* up to a positive (negative) extremum, then decreases (increases) to the negative (positive) infinite-horizon limit.

Despite the system’s complexity, the above results have the following consequences, that include our secondary results.

**Figure 3:**
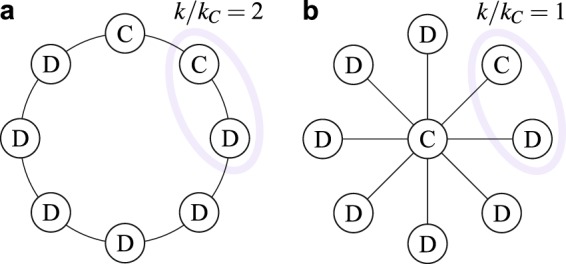
Direct reciprocity and model-predictive rationality in regular and heterogeneous networks. Consider (**a**) the ring ($k=2$ for all nodes) and (**b**) the star network ($\langle k\rangle \simeq 2$ for large *N*) in the infinite-horizon limit. The ring-${R}_{{\rm{fix}}}^{\infty }$ is given by (1) with $k/{k}_{{\rm{C}}}=2$ and a single D drives the population to all-D if $r < {R}_{{\rm{fix}}}^{\infty }$. The star-${R}_{{\rm{fix}}}^{\infty }$ is much higher, because the ratio *k*/*k*_C_ peaks at *N* − 1 for the central node with only one C-neighbor. However, if the central node is a C, the D-leaves will change to C under the weakest requirement on *r* ($k/{k}_{{\rm{C}}}=1$ in (1)) and there are high chances that this occurs before the central node revises her strategy. (The probability that a D-leaf revises before the central node is given by the formula at point (ix), in which *k* is replaced by the number of D-leaves.) If the number *k*_C_ of C-leaves raises to satisfy condition (1), the population then evolves to all-C, and this occurs with probability higher than 1/2 starting with no C-leaves and *r* equal to the ring-${R}_{{\rm{fix}}}^{\infty }$ (the probability goes as 1/2 + 1/(2*N*) for small *δ*, see SI Sect. S9). That is, on average, the isolated central C drives the star to all-C under a milder condition on *r* w.r.t. the ring with some initial D’s.

(i) The state all-C is an equilibrium of our model (or better an absorbing state of the Markovian dynamics) under a requirement on *r* that is much milder than the fixation threshold (see condition (1) with ${k}_{{\rm{C}}}=k$).
(ii) The state all-D is always an equilibrium (see condition (1) with ${k}_{{\rm{C}}}=0$), though a coordinated switch to C by a small cluster of players (not considered in our model) can give a payoff gain.
(iii) Isolated C’s (${k}_{{\rm{C}}}=0$) change to D as soon as they revise their strategy. This does not mean that cooperation cannot start from a single C-agent, since D-neighbors could change to C before the isolated C changes to D.
(iv) Indeed, a D player connected to an isolated C can change to C, provided *r* is sufficiently large (see condition (1) with ${k}_{{\rm{C}}}=1$).
(v) Secondary result 1: direct reciprocity ($d > {d}_{{\rm{\min }}}$) is necessary for the evolution of cooperation. With no reciprocity ($d={d}_{{\rm{\min }}}$), the C-strategy is unconditional (${\delta }_{d}=1$ and ${P}_{{\rm{CD}}}^{\infty }=1$ in (1)) and D is the well-known best option for any *r*. Moreover, as ${P}_{{\rm{CD}}}^{\infty }$ gets smaller together with *δ*_*d*_, a stronger reciprocity (larger $d\in ({d}_{{\rm{\min }}},{d}_{{\rm{\max }}})$) makes the fixation threshold (2) milder.
(vi) Secondary result 2 (first part): the multi-step predictive horizon ($h\ge 2$) is necessary for cooperation, because of the above discussion on ${\rm{\Delta }}{\pi }_{{\rm{C}}}^{1}$ and ${\rm{\Delta }}{\pi }_{{\rm{D}}}^{1}$.
(vii) Secondary result 2 (second part): increasing the horizon *h* reduces the threshold *R*_fix_. Indeed, if $r={R}_{{\rm{fix}}}$ for a given *h*, the node with maximal degree gives either ${\rm{\Delta }}{\pi }_{{\rm{C}}}^{h}=0$ and ${\rm{\Delta }}{\pi }_{{\rm{D}}}^{h} > 0$ or ${\rm{\Delta }}{\pi }_{{\rm{C}}}^{h} < 0$ and ${\rm{\Delta }}{\pi }_{{\rm{D}}}^{h}=0$, depending on the composition of its neighborhood. By the behavior of ${\rm{\Delta }}{\pi }_{{\rm{C}}}^{h}$ and ${\rm{\Delta }}{\pi }_{{\rm{D}}}^{h}$ described above, the former decreases while the latter increases by adding one prediction step. With $h+1$ steps, then, all C’s remain C and all D’s changes to C when revising strategy, so that *r* is above threshold.
(viii) The inertia to change also helps cooperation, in the sense that, for a given direct reciprocity *d*, a lower update rate *δ* gives a lower biased rate *δ*_*d*_ (longer abstention periods, on average), and hence a smaller ${P}_{{\rm{CD}}}^{\infty }$ and a milder fixation threshold (2).
(ix) Secondary result 3: our main result probabilistically holds also starting from a single C in the network, provided her degree *k* is not too small. With $r > {R}_{{\rm{fix}}}$, the probability that a D-neighbor changes to C before the initial C changes to D goes as $1-1/(k+1)$ for small *δ* (shown in SI Sect. S9).
(x) Secondary result 4 (first part): according to condition (1) and the fixation threshold (2), and given the network size *N*, cooperation is favored in homogeneous (regular or single-scale) sparse networks, because of the low maximal degree, compared to dense or heterogeneous networks. However, keeping the same average degree, heterogeneous networks have more low-degree nodes compared to homogeneous ones, so that, while isolated C’s might have higher chances to change to D (see point (ix)), their D-neighbors change to C, with similar probability, under milder returns (condition (1)). Overall, network heterogeneity should facilitate the invasion of cooperation and make fixation more demanding, provided the initial C’s are placed at random.
(xi) Secondary result 4 (second part): the comment at point (ix) and two simple examples in Fig. 3 suggest that network heterogeneity helps cooperation if C’s initially occupy the network’s hubs. Essentially, low-degree D’s connected to a C change to C under a mild requirement on *r* (low ratio *k*/*k*_C_ in (1)). However, to have many low-degree D’s connected to few initial C’s, we need high-connected C’s. Hence, especially starting at low levels of cooperation, degree heterogeneity and the placement of the initial C’s in the network’s hubs together reduce the requirement on *r* to fixate cooperation. Compared to imitation update (in which C-hubs need a significant fraction of C-neighbors to persist), our predictive strategy update allows the formation of C-clusters even starting from isolated C-hubs, who pay (or better invest in) the initial cost of exploitation. If however most of the hubs are D’s, network heterogeneity turns harmful to cooperation (see point (x)).

### Numerical results

To quantify the analytical results, we have run many numerical simulations on several networks of $N=1000$ nodes: ring and planar lattices, single-scale (Watts-Strogatz model with full rewiring) and scale-free (Barabási-Albert model) random networks, and the complete (all-to-all) network. The results for 1% initial fraction of (normally reciprocating, $d=0$) C’s on planar 4- and 8-neighbor lattices and on random networks with average degree $\langle k\rangle =4$ and 8 are reported in Fig. 2. See SI Fig. S1 for 50% initial C’s and S2 for a degree-4 ring lattice (a ring of nodes each connected to the 4 nearest nodes in the loop) and the complete network; see also SI Fig. S3 for the case of super/sub-normal reciprocity (*d* positive/negative) and for different values of the update rate *δ*. For random networks, we have separately simulated the random placement of the initial C’s and the placement according to degree-ranking.

The simulations confirm that cooperation is able to invade, persist, and fixate in any network, provided the PD return *r* is large enough. For any combination of network structure, initialization, and model parameters, the simulations starting from 1% initial C’s identify two thresholds on *r*: a lower threshold *r*_inv_ above which, on average, cooperation invades and persists; an upper threshold *r*_fix_ above which cooperation always invades and fixates. For *r* between *r*_inv_ and *r*_fix_, the average asymptotic fraction of C’s (solid lines) is not representative of the level of cooperation one should expect in a single simulation (dots), as cooperation disappears/fixates in most of the cases (see dots at fractions 0 and 1) and typically settles at low C-levels (below 20%) in the rest of the cases. A deeper analysis of the simulations of Fig. 2 not ended in all-C or all-D (the dots in the open interval (0, 1)) indeed reveals that most of dots above 0.2 denote simulations that converge to all-C on a longer timescale, whereas dots below 0.2 typically represents simulations ending in a nontrivial stalemate—an equilibrium different from all-C and all-D—or showing long-term fluctuations (see SI Sect. S10 for examples of nontrivial stalemates and fluctuations in the simple network of Fig. 1b; Sects S11 and S12 for further details on networks and numerical simulations).

Note that the threshold *r*_fix_ is much smaller than the theoretical *R*_fix_ of our main result (see the average $\langle {\bar{R}}_{{\rm{fix}}}\rangle$ over the simulations of a given type in Fig. 2, where ${\bar{R}}_{{\rm{fix}}}$ is the upper bound to *R*_fix_ derived in SI Sect. S7). This is due to the stochastic effect described at point (ix). Essentially, even if some of the C’s initially need a higher *r* to remain C, by the time they revise their strategy the *r*-gap could have vanished because of D-to-C changes in the neighborhood. This overcompensates the opposite effect due to the fact that *R*_fix_ is computed starting from a cluster of two C’s, whereas initial C’s are most often isolated (except for scale-free networks with degree-rank-C-placement, because hubs are likely connected among themselves; see the average % for each panel in Fig. 2). Starting with random pairs of connected initial C’s indeed results in lower *r*_inv_ and *r*_fix_ (shown in SI Fig. S4).

As expected from the arguments at points (vii) and (x), the thresholds *r*_inv_ and *r*_fix_ decrease if the horizon *h* is extended and increase with the network’s average degree, given all other details (Fig. 2: compare the different colors within each panel and left vs right panels). Both effects are weakened, as expected, starting from higher initial C-levels (shown in SI Fig. S1).

Degree heterogeneity also works as theoretically predicted. It works against the fixation of cooperation under random placement (Fig. 2: compare single-scale and scale-free networks and note the significantly higher *r*_fix_ in the latters; *r*_inv_ is slightly lower, as predicted at point (x)). The effects are again weakened starting from higher initial C-levels (SI Fig. S1). Placing the initial C’s in the network’s hubs does favor cooperation, both in terms of invasion and fixation (Fig. 2: compare single-scale and scale-free networks under degree-rank-C-placement and note the lower *r*_inv_ and *r*_fix_ in scale-free networks; also note that the type of placement is irrelevant for single-scale networks). The effect is however moderate.

Finally, we have checked the robustness of the above results with respect to a different type of scale-free network (with tunable transitivity, Holme-Kim model, see SI Fig. S5), and by limiting the agents’ computational skills and rationality (see SI Figs S6 and S7, respectively).

## Discussion

We have designed and analyzed an EGT model to validate two specific hypotheses: (1) that reciprocal cooperation can evolve on social networks, against unconditional defection, under a process of strategy update that rationally pursues the individual interest; and (2) that this EGT setup shows network reciprocity. Our motivation originates from a mismatch between theoretical predictions and experiments with human subjects. Network reciprocity—the beneficial effect on cooperation possibly emerging when agents’ interactions are limited by a sparse network of contact—has been theoretically investigated with models based on imitative processes of strategy update, with the result that cooperation has better chances to fixate, the fewer are, on average, the connections between the agents. This ‘rule’ of network reciprocity, precisely the condition $r > \langle k\rangle$, where *r* is the economic return of the game interaction and $\langle k\rangle$ is the network’s average degree, is consistent with all available experiments^45–47,51,52,55–58^, in which however the subjects’ behavior proved incompatible with the assumed imitation process. Indeed, when the interaction is far from all-to-all, imitating a better performing neighbor gives no guarantee to increase our own payoff, and models implementing the simplest rational rule to do what is best for us in the next interaction—the best response update—show no network reciprocity^53^. Our results reconcile the mismatch, showing network reciprocity in an EGT setup that is more adherent to the common rationale and to the behavioral traits emerging from experiments.

Our model does not specifically describe any of the available experimental setups. The link to the experiments is in the choice of the two strategies we confront: a C-strategy implementing a form of direct reciprocity and the benchmark D-strategy of unconditional defection. The analysis of some of the experiments (in which, as a rule of the experiment, subjects take one decision per game round to cooperate or defect with all neighbors) indeed revealed three general facts^45,47,48,50^: the first is that subjects’ behavior is typically biased by previous decisions (cooperation being more/less likely in subjects who cooperated/defected in the previous round), so that a cooperative or defective ‘mood’ can be identified. The second is that, while in the C-mood, subjects reciprocate cooperation (cooperation being more/less likely the higher/lower was the number of cooperating neighbors in the previous round); third, subjects in the D-mood defect rather unconditionally.

The main novelty of our model is the predictive rule for strategy update, that extends the best response rule to an horizon of future interactions. It is a modeling assumption intentionally not grounded on experimental evidence; it is our way to introduce rationality in the model. Identifying the update rule from experiments is in any case a difficult task^46,48,50,57^ and, somehow, an ill-posed problem. The neat distinction between strategies and strategy update is actually pertinent to models only; e.g., the observed moody behavior has been also described as a unique strategy conditioned by the agent last decision^48^. Moreover, different subjects can use (or learn) different rules and the result is likely to depend on the experimental setting. Although the average rates at which subjects changed between the C and D moods have been estimated from data^45–48,50^, and turned out independent on the network structure^46^, this does not unveil the decision-making process.

Note that the novelty is not strictly in the use of a non-imitative, also called *innovative*, rule for strategy update, as the best response is itself innovative. Moreover, there has recently been a growing interest for innovative rules^59,60^ and for the coexistence^61^ and competition^62^ between imitative and innovative rules. So far, however, no innovative update showed any significant network effect on cooperation^53,59^, at the point that network reciprocity is often considered a feature of imitative dynamics only^61^. The novelty is hence to show network reciprocity under an innovative evolutionary process.

The way in which we implement direct reciprocity—allowing C’s to selectively abstain from playing the PD with D-neighbors—also brings some novelty. Optional participation is known to relax social dilemmas when a baseline payoff is granted to loners^19,63,64^. However, abstention has often been considered an independent strategy^63–65^, or a probabilistic option for both C’s and D’s^66^, rather than an option for cooperators to lower exploitation risk (as proposed in ref.^19^ in a non-evolutionary setting). This is the option that makes abstention a form of direct reciprocity. Differently from previous models, we assign no payoff to such an option. We prefer abstention rather than retaliation—cooperators defecting exploiters—because this is more connatural to the C-mood. Although there is typically no difference in a single round (because both abstention and mutual defection give no payoff), abstaining C’s communicate their mood to exploiters, who will take it into account when revising their strategy. This is because we assume that agents who agree to play see whether the opponent agrees or abstain, whereas abstaining agents get no information. Of course our model does not represent situations in which the interaction is blind and agents only see their own payoff.

Direct reciprocity deserves another comment. It requires repeated interactions among the same agents, as well as cognitive abilities to recognize individuals and remember past interactions. Originally^1,2^, it has been studied in iterated games, i.e., (non-evolutionary) games involving only two players (rather than a population of two types of players) who know the probability $w > 0$ of a next interaction. In EGT, there are two ways in which one can study direct reciprocity: either the single game round consists of an iterated game between each pair of neighbors—an option allowing direct reciprocity in any network, so far investigated in the socio-economic context under imitative strategy update^32,67^—or the single game is one-shot, but agents repeatedly interact on a static (or slowly changing) sparse network, as we do. Indeed, the unchanging network grants a next round, while the sparsity of the connections makes cognitive tasks affordable. Of course hubs need more resources than leaves, but this is typically built-in in the socio-economic structure of the society.

The other elements of our model are rather standard (see Methods for full detail). We now discuss our results and their scope in the light of previous theoretical and empirical work. First of all, the interplay between direct and network reciprocity has been already addressed^32,67^ with models that, in the socio-economic context, are however based on imitative strategy update. The general result is that direct reciprocity in the C-strategy favors cooperation in synergism with the population structure, the latter being defined by a network^32^ or through assortative encounters^67^. Our model confirms the role of direct reciprocity in triggering the positive network effect on cooperation, and does it under a rational process of strategy update.

We actually show that direct reciprocity, or some other mechanism supporting cooperation (see the Introduction), is necessary to rationally avoid the dominance of defection in a networked PD, and hence to see any network effect. Focusing on direct reciprocity, we expect similar results by rationally confronting unconditional D with any reciprocating form of conditional cooperation. To test this claim, we have run preliminary simulations on the famous tit-for-tat strategy^2^ (traditionally used to model direct reciprocity, including refs^32,67^). Worth of note is that our model highlights a double role of static and sparse networks of contacts. Not only these are the structures that favor cooperation according to our model, but these are, as well, the structures that make our model feasible, because the cognitive tasks required by both direct reciprocity and model-predictions scale with the size of the agent’s neighborhood. We did use our model on a large complete network as well, but we did it for benchmarking purposes, to set the case to compare with in looking for network effects.

Should we then expect to see a decline of human cooperation in real-world socio-economic systems that are arguably getting more and more connected? This seems to be the take-home message from network reciprocity, that our model even made more convincing. Models and experiments showing network reciprocity were however designed to isolate the effect of static network of contacts. In real-world systems, other mechanisms can support cooperation despite the high interconnection, such as cultural, economic, and political agreements, or punishment mechanisms; the latter, in particular, has been recently shown to inhibit the network effect in experiments^52^. Moreover, real-world networks are often dynamic. In particular, adaptive networks, in which agents can cut links and establish new ones as a result of the game interaction, proved to be cooperation promoters, with both theoretical support^65,68,69^, however based on imitation update, and empirical evidence^70–72^ (see ref.^73^ for a review). Note that allowing C-agents to cut links with exploiters and seek for new cooperators is an exit option^18,54^ similar to the one we use; we have a static topology of connection, but links are temporarily inhibited. Interestingly, adaptive rewiring can be feasible even in medium-large neighborhoods, as, e.g., random rewiring does not involve intense cognitive tasks. It would be then interesting to study adaptive rewiring under model-predictive update, to see whether it supports the evolution of cooperation in dense networks.

Two aspects on which our results significantly differ from those obtained with imitative update concern the invasion potential of cooperation and the role played by the degree heterogeneity of the network. About the first aspect, we have shown (analytically and quantified numerically) the existence of a threshold on the game return *r* (scaling with the network connectivity) above which cooperation has high chances to fixate starting from a single C-agent, i.e., chances of the order $1-1/(k+1)$ at a low rate of strategy update, where *k* is the degree of the initial C (see analytical result (ix)). This is different from what is granted by imitative update under the condition $r > \langle k\rangle$, i.e., the fixation probability—the probability that cooperation fixates starting from a single randomly placed C—being larger than 1/*N* in a network with *N* nodes, 1/*N* being the fixation probability under a totally random process of strategy update. When $r > \langle k\rangle$ is weakly satisfied in a large network, cooperation almost surely disappears starting from a single C (probability 1 − 1/*N*). To have higher chances of fixation, a significantly larger *r* is typically required and, especially when selection is strong (SI note 4), cooperation cannot invade anyhow. Consider, e.g., a single C in the lattice of Fig. 1a. If selection is strong, the C most likely imitates a D-neighbor as soon as she revises her strategy, whereas D-neighbors do not imitate the C. The probability of invasion—to go from one to two C’s—is negligible after each game round, whereas the C sooner or later switches to D. We hence conclude that imitative strategy update does not support the invasion of cooperation. Not surprisingly, most theoretical studies based on imitation^27–43^ consider random initial conditions with 50% C’s (33% in ref.^53^). We have, e.g., tested unconditional C against unconditional D playing the PD under the pairwise comparison imitation rule with strong selection (the one used in the majority of the above mentioned works; see SI notes 3 and 4). Starting from 1% initial C’s on the same network structures of Fig. 2, cooperation systematically disappeared up to *r* = 5000, except for scale-free networks with degree-rank-C-placement (where C-hubs are known to form clusters) in which we found invasion only for *r* larger than 20.

About network heterogeneity, our model helps to clarify another mismatch between theory and experiments. From the theoretical side, a considerable effort has been devoted to identify the network structures that best favor the evolution of cooperation (unconditional C against unconditional D) under imitative update^27–29,31^, with the general answer that, for given (sufficiently small) average connectivity, heterogeneous networks—e.g., scale-free networks—do better than homogeneous networks—lattices and single-scale networks. Indeed, a C-hub (individual *i* with degree ${k}_{i}\gg \langle k\rangle$) with a significant fraction (say 50%) of C-neighbors is copied by a low-connected D-neighbor *j* under a mild requirement on the PD return *r* (payoff per game round: ${\pi }_{i}=(r-1){k}_{i}/2-{k}_{i}$; ${\pi }_{j}=r$ if *j* has no other C-neighbors). C-hubs can then build C-clusters, whereas this requires higher returns in homogeneous networks (*k*_*i*_, ${k}_{j}\simeq \langle k\rangle$). On the contrary, the network structure played a marginal role in experiments^47,48^, as well as in our model. Our results show two weak opposite effects of network heterogeneity on the fixation of cooperation, depending on whether the initial C’s are placed randomly in the network or according to the degree rank. In the first case, especially starting at low initial level of cooperation, the network’s hubs are most likely D’s and their low-connected D-neighbors require high returns to change to C, provided they have a C-neighbor. In contrast, C-hubs attract low-connected D-neighbors in changing to C under mild game returns. Under a slow strategy update, the number of C-neighbors of a C-hub can raise, while the hub pays the cost of building the cluster. In other words, initially isolated C-hubs invest in the future establishment of cooperation. Moreover, hubs are likely connected among themselves, so that placing the initial C’s in the network’s hubs forms clusters of C’s that mitigate the investment. Network heterogeneity therefore turns beneficial to cooperation under this strategic placement of the initial C’s.

We conclude by going back, once more, to the two key elements of our model: direct reciprocity and the model-predictive strategy update. The first is taken because of the empirical evidence, the second is assumed to rationally drive evolution by self-interest. Both proved necessary to sustain cooperation and to show network reciprocity. Under the rational tenet of selfishness, we claim that a mechanism supporting cooperation, direct reciprocity in the first place, and a predictive horizon that goes beyond the next interaction are the two necessary and sufficient ingredients to explain cooperation over social ties. Besides their conceptual value, we believe our findings can inspire further experimental work. Our model-predictive strategy update is not an easy one to imagine in real-world networks, because of the nontrivial cognitive tasks involved and because humans might not be as rational as we assume. However, we believe it could approximatively emerge as the result of an intuitive, rather than computational, human behavior. Indeed, our results proved to be robust to significant error rates in computing model-predictions and to significant degrees of irrationality (see Numerical results). But most importantly, we envisage the design of new experiments. For example, to confirm that humans do have a C or D mood, it is important to allow them to temporarily abstain from playing with specific neighbors, to avoid confounding risk-avoiding defections with a D mood. Allowing independent actions for every neighbor, as we partly do by means of our abstention mechanism, is also important, e.g., to identify a mix of C and D as the absence of a mood. This definitely poses experimental challenges^70^, and it has been recently shown to enhance cooperation in static networks^74^.

## Methods

### The model

We consider a connected static network of *N* nodes, each occupied by an agent of the population. The pairwise game interaction between agents proceeds in discrete rounds. At each round, each agent is a C or a D strategist (C or D agent), she accordingly plays the game with all her neighbors, and collects payoffs. After each round, each agent independently decides whether to revise her strategy with probability *δ*, assumed, for simplicity, uniform across the population (the process of strategy update is further detailed at the end of this section).

In each game interaction, C-agents have two options, abstain from playing (option A) or cooperate (option C); by abstaining, they do not see the opponent’s choice; D-agents always play and defect (option D). If both opponents agree to play, the game is a PD (with return *r*); otherwise no one gets or looses anything, resulting in the following payoff matrix:3

The matrix gives the payoffs for the row agent; those for the column agent are given by the transposed matrix. The shading of the first row indicates that those zero payoffs are indistinguishable by an abstaining C-agent, whereas, a D-agent (third row) is able to distinguish between abstaining C and D neighbors.

The abstention mechanism implements a form of direct reciprocity between C-agents. After getting exploited by the neighbor *j*, the C-agent *i* decides to abstain at the next game round with the probability 1 − *δ* that *j* has not revised strategy. Similarly, after *t* − 1 consecutive abstentions, *i* opts for another one with the probability (1 − *δ*)^*t*^ (vanishing for increasing *t*) that *j* has not revised since having exploited *i*. This is the behavior of ‘normally’ reciprocating C-agents (normal reciprocity). ‘Super/sub’-reciprocating C’s abstain for longer/shorter periods, on average (super/sub-normal reciprocity). They formally behave as normally reciprocating ones, but base their decisions on the under/over-biased update rate *δ*_*d*_ defined in (1), where −100*d*% is the negative/positive bias, also assumed uniform across the population. Parameter $d\in ({d}_{{\rm{\min }}}=-\,(1/\delta -1),{d}_{{\rm{\max }}}=1)$ modulates the strength of direct reciprocity: no reciprocity at $d={d}_{{\rm{\min }}}$; sub-normal reciprocity for $d\in ({d}_{{\rm{\min }}},0)$; normal reciprocity at $d=0$; super-normal reciprocity for $d\in (0,{d}_{{\rm{\max }}})$; extreme reciprocity at $d={d}_{{\rm{\max }}}$ (the extreme cases $d={d}_{{\rm{\min }}}$ and $d={d}_{{\rm{\max }}}$ are considered unadmissible in our model). The resulting distribution of the length *a* of abstention periods, i.e., the probability of *a* consecutive abstentions followed by a cooperation, is

4 $${\rm{Prob}}(a)=\{\begin{array}{ll}{\delta }_{d} & {\rm{if}}\,a=0,\\ \prod _{t=1}^{a}\,{(1-{\delta }_{d})}^{t}(1-{(1-{\delta }_{d})}^{a+1})={(1-{\delta }_{d})}^{a(a+1)/2}(1-{(1-{\delta }_{d})}^{a+1}) & {\rm{if}}\,a > 0.\end{array}$$

**Figure 4:**
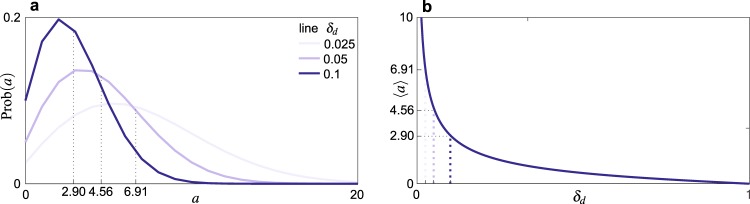
The statistics of abstention periods. (**a**) The distribution Prob(*a*) of Eq. (4). (**b**) The mean $\langle a\rangle$ of the distribution as a function of its parameter *δ*_*d*_.

It is graphed in Fig. 4 for different values of the reciprocity-biased update rate *δ*_*d*_, together with the *δ*_*d*_-dependence of its mean.

We implement our model by endowing each agent *i* with the set of probabilities *p*_*ij*_’s, $j=1,\ldots ,N$, that *i* will agree to play with *j* at the next game round. Initially, ${p}_{ij}={p}_{ji}=1$ if *i* and *j* are neighbors, while ${p}_{ij}={p}_{ji}=0$ otherwise; the $N\times N$ matrix $P=[{p}_{ij}]$ hence defines the network topology. At each round, the $(i,j)$ PD interaction takes place with probability *p*_*ij*_*p*_*ji*_, i.e., only if both players agree to play. When the C-agent *i* gets exploited by neighbor *j*, she sets ${p}_{ij}={p}_{1}={\delta }_{d}$. If *i* decides not to play with *j* at the next round, *p*_*ij*_ is updated to ${p}_{2}=1-{(1-{\delta }_{d})}^{2}$; after *t* − 1 consecutive abstentions, the probability to play at the next round is

5 $${p}_{t}=1-{(1-{\delta }_{d})}^{t},\,t\ge 1,$$

i.e., the probability (increasing to one with *t*) that *j* has revised at least once since having exploited *i*. As soon as *i* decides to play, *p*_*ij*_ is reset to *p*_1_ if *j* defects again, otherwise it is set to ${p}_{0}=1$ to reciprocate cooperation. D’s always have ${p}_{ij}=1$ toward all neighbors. We therefore implement a static network topology with dynamic weights (the probabilities *p*_*ij*_) associated to the connections.

Strategy update is asynchronous^75^, with rate $\delta \in (0,1)$ (per game round) uniform across the population, i.e., after each game round (also following the update of the probabilities *p*_*ij*_), each individual independently decides whether to revise her strategy with probability *δ*. C’s who revise compute the payoffs they expect to collect if remaining C, ${\pi }_{{\rm{CC}}}^{h}$, or behaving as D, ${\pi }_{{\rm{CD}}}^{h}$, over the next *h* rounds, and change to D if the expected gain ${\rm{\Delta }}{\pi }_{{\rm{C}}}^{h}={\pi }_{{\rm{CD}}}^{h}-{\pi }_{{\rm{CC}}}^{h}$ is positive. Similarly, D’s who revise compute their expected collected payoff ${\pi }_{{\rm{DC}}}^{h}$ and ${\pi }_{{\rm{DD}}}^{h}$ and change to C under a positive expected gain ${\rm{\Delta }}{\pi }_{{\rm{D}}}^{h}={\pi }_{{\rm{DC}}}^{h}-{\pi }_{{\rm{DD}}}^{h}$. Future payoffs are not discounted because of the short predictive horizon *h* (see Model parameters below). When changing to D, the C-agent *i* sets ${p}_{ij}=1$ toward all neighbors. When changing to C, D-agent *i* sets ${p}_{ij}={p}_{1}={\delta }_{d}$ toward her D-neighbors, to possibly avoid being exploited at the next round. Note that, by construction, either ${p}_{ij}=1$, or ${p}_{ji}=1$, or both.

The payoff predictions are computed assuming that neighbors behave according to the model society here described. The information available to agent *i* on her *j* neighbor’s state (strategy and probabilities *p*_*ji*_’s) only comes from the pairwise interactions and is summarized in the sets of probabilities *p*_*ij*_’s and *p*_*ji*_’s. Indeed, for agent *i*, remembering the last $(i,j)$ PD interaction, i.e., when it took place and the outcome, is equivalent to update both sets of probabilities, according to the assumed abstention mechanism. For the revising C-agent *i*, ${p}_{ij} < 1$ implies that *j* exploited *i* at the last $(i,j)$ PD interaction; otherwise ${p}_{ij}=1$ and *i* and *j* both cooperated at the last round if ${p}_{ji}=1$ or *j* did not play because exploited by *i* at the last $(i,j)$ PD interaction if ${p}_{ji} < 1$. Agent *j* is considered a D in the first case, even though she might have changed to C since *i* and *j* last played the PD; *j* is correctly considered a C in the second case. For the D-agent *i*, ${p}_{ji} < 1$ implies *i* exploited *j* at the last $(i,j)$ PD interaction; otherwise ${p}_{ji}=1$ and *i* and *j* both defected at the last round. In both cases, agent *j* is correctly identified as C and D, respectively. Thus, only D-agents have full information on their neighbors’ state, whereas C-agents can underestimate the number of their C-neighbors.

Payoff predictions are accordingly computed in SI Sects S4 and S5, disregarding, for simplicity, possible changes in the neighbors’ strategies. To this end, two probabilities are first computed in SI Sects S2 and S3 for the revising C-agent *i*, with ${p}_{ij}={p}_{{t}_{ij}}$ for some integer *t*_*ij*_, to play the PD with the neighbor *j*, with ${p}_{ji}={p}_{{t}_{ji}}$ for some integer *t*_*ji*_: the probability ${P}_{{\rm{CD}}}^{t}({t}_{ij})$, ${t}_{ij}\ge 1$, to play with the D-neighbor *j* at round *t* of the predictive horizon; and, similarly, the probability ${P}_{{\rm{CC}}}^{t}({t}_{ji})$, ${t}_{ij}=0$, ${t}_{ji}\ge 0$ to play with the C-neighbor *j*. They can both iteratively be computed with the following recursions:

6a $${P}_{{\rm{CD}}}^{t+1}({t}_{ij})={P}_{{\rm{CD}}}^{t}({t}_{ij}){\delta }_{d}+(1-{P}_{{\rm{CD}}}^{t}({t}_{ij}))\,((1-{\delta }_{d}){P}_{{\rm{CD}}}^{t}({t}_{ij})+{\delta }_{d}),$$

6b $${P}_{{\rm{CC}}}^{t+1}({t}_{ji})={P}_{{\rm{CC}}}^{t}({t}_{ji})+(1-{P}_{{\rm{CC}}}^{t}({t}_{ji})){p}_{{t}_{ji}+t},$$

initialized at ${P}_{{\rm{CD}}}^{1}({t}_{ij})={p}_{{t}_{ij}}$ and ${P}_{{\rm{CC}}}^{1}({t}_{ji})={p}_{{t}_{ji}}$ (${P}_{{\rm{CD}}}^{t}$ converges, as $t\to \infty$, to the infinite-horizon limit ${P}_{{\rm{CD}}}^{\infty }$ of condition (1), independently of initialization). The resulting formulas for the expected payoff gains ${\rm{\Delta }}{\pi }_{{\rm{C}}}^{h}$ and ${\rm{\Delta }}{\pi }_{{\rm{D}}}^{h}$ are

7a $${\rm{\Delta }}{\pi }_{{\rm{C}}}^{h}=-\,r\,\sum _{{p}_{ij}=1}\,\mathop{\underbrace{(\sum _{t=1}^{h}\,{P}_{{\rm{CC}}}^{t}({t}_{ji})-\sum _{t=1}^{h}\,{P}_{{\rm{CD}}}^{t}({t}_{ji}))}}\limits_{ > 0\,{\rm{for}}\,h\ge 2}+\sum _{{p}_{ij}=1}\,\sum _{t=1}^{h}\,{P}_{{\rm{CC}}}^{t}({t}_{ji})+\sum _{{p}_{ij} < 1}\,\sum _{t=1}^{h}\,{P}_{{\rm{CD}}}^{t}({t}_{ij}),$$

7b $${\rm{\Delta }}{\pi }_{{\rm{D}}}^{h}=r\,\sum _{{p}_{ji} < 1}\,\mathop{\underbrace{(\sum _{t=1}^{h}\,{P}_{{\rm{CC}}}^{t}({t}_{ji})-\sum _{t=1}^{h}\,{P}_{{\rm{CD}}}^{t}({t}_{ji}))}}\limits_{ > 0\,{\rm{for}}\,h\ge 2}-\sum _{{p}_{ji} < 1}\,\sum _{t=1}^{h}\,{P}_{{\rm{CC}}}^{t}({t}_{ji})-\sum _{{p}_{ji}=1}\,\sum _{t=1}^{h}\,{P}_{{\rm{CD}}}^{t}(1).$$

### Model parameters

The model parameters are summarized in Table 1, together with their reference values and the perturbed values used in the numerical analysis. The size *N* and the matrix $P=[{p}_{ij}]$ define the network of the agents’ connections; parameters *r*, *δ*, *h*, and *d* set the dynamics of our model; $\langle a\rangle$ and *δ*_*d*_ are derived quantities. Though we do not have a closed formula for $\langle a\rangle$ as a function of *δ*_*d*_, there is a one-to-one correspondence (graphically expressed by Fig. 4b) between the two quantities. Either *d*, *δ*_*d*_, or $\langle a\rangle$ can therefore be used as a model parameter, the other two being accordingly derived for any given *δ*.

We consider a slow strategy update (small *δ*), compared to the discrete time of game rounds. This allows to disregard the neighbors’ updates in computing the payoff predictions over relatively short predictive horizons. In our simulations we have limited the product *δh*—upper bounding the neighborhood fraction possibly subject to change within the horizon—to 0.3 (e.g., $\delta =0.05$ and $h\le 5$ in our reference setting of Fig. 2).

We consider significant cases of super- and sub-normal reciprocity: $d=1/2$ and $d=-\,1$ corresponding to a −50% and +100% bias of the update rate used in the statistics of the abstentions. The corresponding average lengths $\langle a\rangle$ of abstention periods is reported in Table 1.

### Networks structures and numerical simulations

See Numerical analysis. See SI Sects S11 and S12 for further details.

## Supplementary information

SI
