# Supplementary material for: Direct reciprocity and model-predictive rationality explain network reciprocity over social ties
Source: Sci Rep. 2019 Apr 1;9:5367. doi: 10.1038/s41598-019-41547-w (PMC6443768; doi:10.1038/s41598-019-41547-w)
Supplement: Supplementary file 1 — SI [file 41598_2019_41547_MOESM1_ESM.pdf]

Supplementary Information

Direct reciprocity and model-predictive rationality explain network reciprocity over social ties

Fabio Dercole[1,*], Fabio Della Rossa[1], and Carlo Piccardi[1]

[1]Department of Electronics, Information, and Bioengineering, Politecnico di Milano, Piazza Leonardo da Vinci 32, I-20133, Milano, Italy
[*]fabio.dercole@polimi.it

Supplementary Notes

1. The four payoffs of the PD are (largest-to-smallest): the *temptation* T to exploit a cooperator, the *reward* R for mutual cooperation, the *punishment* P for mutual defection, and the *sucker* S got by cooperators when exploited; The dilemma is that the best option for each player is to defect, independently on the opponent's choice (because $T > R$ in case the opponent cooperates; $P > S$ in case she defects), so that both get P while they could get $R > P$ by mutual cooperation; moreover the constraint $2R > T + S$ imposes the highest welfare for the pair on mutual cooperation, so to avoid the possibility for the two opponents to agree on sharing the payoffs of an arranged exploitation. We use the standard one-parameter formulation[5–7] $T = r$, $R = r - 1$, $P = 0$, $S = -1$, where $r = b/c > 1$ is the benefit-to-cost of the interaction. In this case, the cost c is on the cooperator while the benefit b goes entirely to the opponent, reason for which this PD is also called *donor-recipient* game. Economically, r it is the return of the interaction. From a social point of view, r measures the 'weakness' of the dilemma; the temptation to defect indeed disappears as $r \to \infty$, while it grows as $r \to 1$. The 'strength' of the dilemma is accordingly measured by $1/(r-1)$. Two standard two-parameter formulations involve T and S or $D_{\rm g} = T - R$ and $D_{\rm r} = P - S$, for given R and P (typically $R = 1$ and $P = 0$), where $D_{\rm g}$ and $D_{\rm r}$ respectively measure two different dilemma strengths: the temptation to 'gamble' on others' cooperation and the 'risk' of cooperating.[76] More precisely, the dilemma strengths are quantified by the scaled parameters $D'_{\rm g} = (T-R)/(R-P)$ and $D'_{\rm r} = (P-S)/(R-P)$, obtained by normalizing $D_{\rm g}$ and $D_{\rm r}$ by the payoff surplus of mutual cooperation over mutual defection. Back to our formulation, we have $D'_{\rm g} = D'_{\rm r} = 1/(r-1)$, so equal strength for temptation and risk.

2. Besides the prisoner's dilemma (PD), three other paradigmatic social dilemmas are used to discuss cooperation among self-interested agents: the *chicken* (CH) (also known as *snowdrift*), the *stag hunt* (SH), and the *harmony* (HA) games.[5–7] Granted that mutual cooperation pays more than mutual defection in all cases, the four games are respectively characterized by the sign pairs $(++)$, $(-+)$, $(+-)$, $(--)$ of the scaled strengths of temptation and risk $D'_{\rm g} = (T-R)/(R-P)$ and $D'_{\rm r} = (P-S)/(R-P)$ (see note 1 for payoff names and parameterizations). That is, temptation to defect and risk to cooperate are both present in the PD, while only the first, only the second, or none of the two is present in the other dilemmas. Temptation and risk are considered the two major distinctive features of social dilemmas, so that the four games cover all combinations, from the harshest dilemma, the PD, to the absence of a dilemma, the HA. Accordingly, the scaled strengths $D'_{\rm g}$ and $D'_{\rm r}$ have shown a universal power in determining the Nash equilibrium of a one-shot game, or the equilibrium fraction of cooperators and defectors in a structured population, under a variety of EGT setups[77] (essentially based on imitative strategy update, see note 3).

3. Several rules for strategy update implement imitative (also called 'non-innovative') evolutionary processes.[6,7] In biology, the *Birth-Death* (DB) and *Death-Birth* (DB) rules are the most used. The first prescribes that an individual is selected to reproduce with probability proportional to her fitness, one of the neighbors is selected to die with uniform probability, and the newborn, a copy of the parent, occupies the empty site. In the second, an individual is selected to die with uniform probability over the whole population, one of the neighbors is selected to reproduce with probability proportional to fitness, and the newborn occupies the empty site. Fitness is a measure of performance in the underlying game; it can simply be the game payoffs collected in the last round (see note 4). (See Ref. 43 for other biologically-inspired evolutionary processes, in which selection acts globally or locally on both birth and death with

possibly independent dispersal and interaction networks.) Both rules are non-innovative because equivalent, in the socio-economic context, to the strategy update of the 'dying' individual, who copies, instead of dying, the strategy of a neighbor. However, only the DB is considered truly imitative, because all individuals have the same chances to be selected for update, whereas individuals connected to better performing ones have higher chances in the BD. The *Imitation* (IM) rule is indeed very similar to DB. It only differs by the fact that the strategy of the updating individual is included in the set of strategies to chose from, so that she has the chance to stay on her own strategy, proportionally to fitness. Another rule used in the socio-economic context is the *Pairwise Comparison* (PC): the first randomly selected agent randomly selects a neighbor and stays or copies the neighbor's strategy proportionally to fitness difference. Variants of the above three rules can be obtained by shaping the mapping from fitness to probability to copy. E.g., the most used rule is PC with probability to copy linearly increasing only with positive fitness difference. It is called *Replicator* rule, because the resulting evolutionary dynamics on the complete network was proved to converge, for large populations, to the well-known replicator dynamics of EGT.[5–7] Also often used is the so-called *Fermi* rule, the PC with probability to copy given by the Fermi exponential function of the fitness difference (going from zero to one, passing from $1/2$ for zero difference, see caption of Fig. S7). It describe decisional errors or irrationality, giving small chances to copy worse performing agents. Another example is the rule *Imitate-the-best*, used by M. A. Nowak & R. May's[22] to first show network reciprocity. It is the IM rule with probability one to copy the fittest among herself and the neighbors. Interestingly, network reciprocity (for unconditional cooperators and defectors playing the PD) has been shown for all the above rules, but BD[24,29] (the condition on the game return r for the fixation of cooperation is the mildest for DB, slightly more demanding for IM and PC, and highly demanding for BD[26]). Essentially, under BD, D's at the boundary of C-clusters reproduce more than boundary C's.

4. Weak selection means that the payoffs of the underlying game contribute to the agent's fitness only to a small extent. It is an interesting limit because it simplifies analytic computations. It represents situations in which the agent's performance is largely determined by factors that are independent of the game interaction and typically assumed time-invariant. These external factors give a baseline fitness to all players, to which the game outcome marginally adds. Selection is strong, as in our model, when the fitness is totally determined by the game outcome. It can simply be the last round payoff, or a function of the payoff that increases more than linearly, so to increase the probability to select the best performance. In the limit of extreme selection[78] the agent with larger payoff is always selected to reproduce or be copied. Note that the selection strength is also controlled by rules for strategy update (see note 3), precisely by the mapping from fitness to probability to copy. E.g., under imitate-the-best, even if selection is very weak, the updating agent copies the neighbor with highest fitness, even though the neighbors' fitnesses are all very similar.

Supplementary Figures

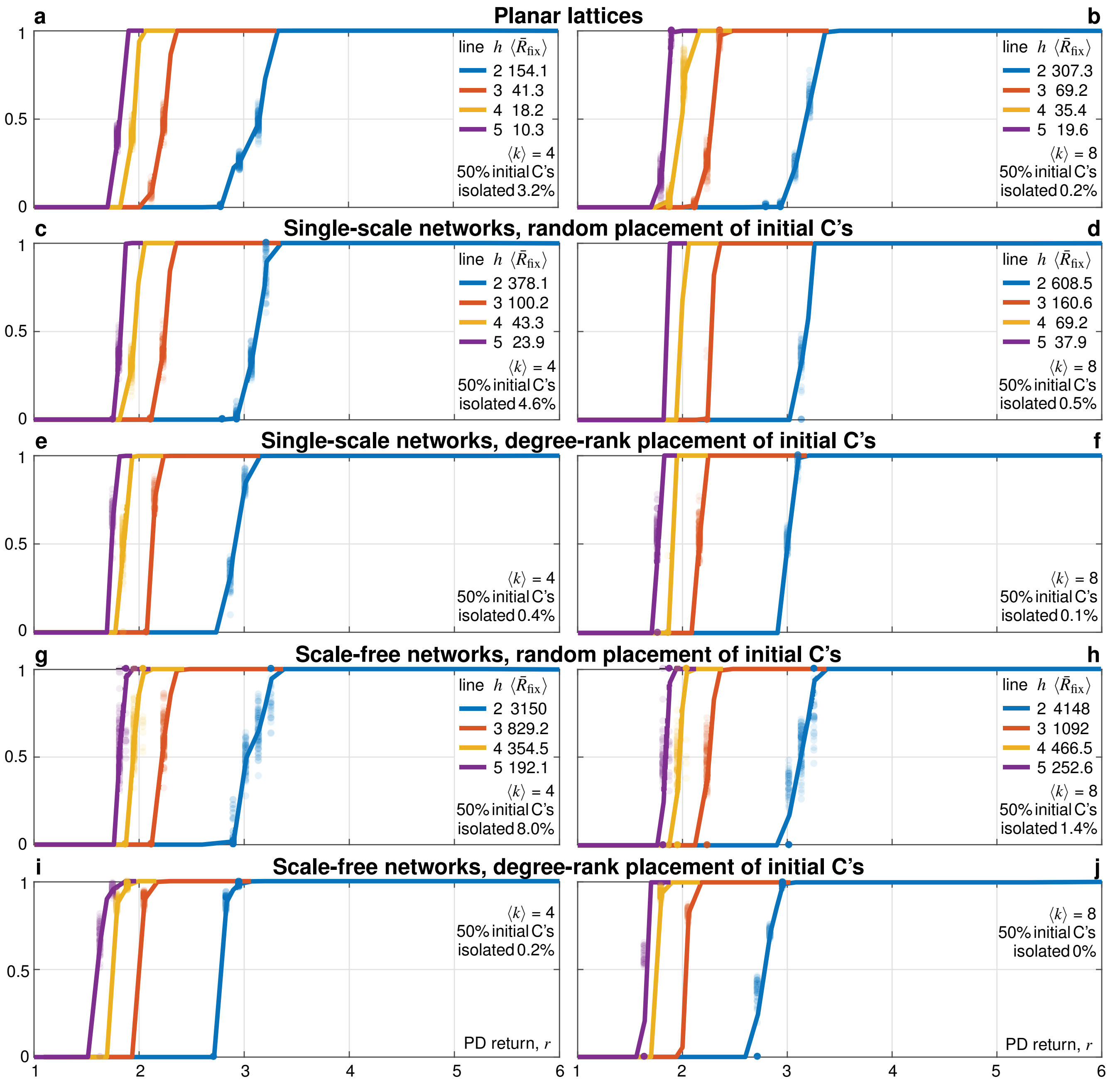

Figure S1. Persistence and fixation of cooperation under direct reciprocity and model-predictive rationality. The figure complements Fig. 2 by showing the results obtained starting from 50% initial C's, all other details unchanged (same model parameters, networks, and initializations). Note that the thresholds on r identified with 50% initial C's have a different meaning w.r.t. the significantly higher r_{inv} and r_{fix} of Fig. 2. Here cooperation persists (resp. fixates), on average, for r above the lower (resp. higher) threshold, being however unable to invade (resp. fixate after invasion) if r is below the r_{inv} (resp. r_{fix}) of Fig. 2. The effects of the different network structures and initializations observed in Fig. 2 are still present but weakened.

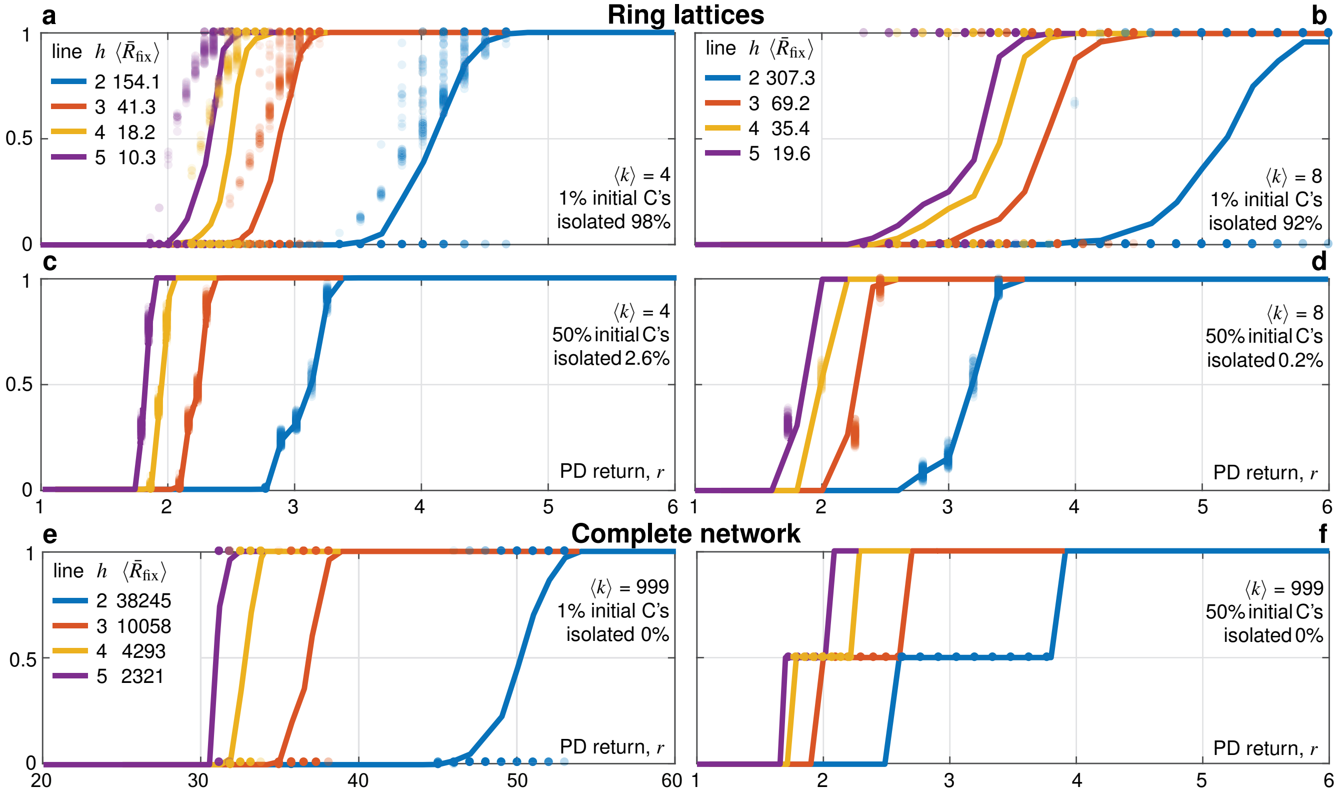

Figure S2. Invasion, persistence, and fixation of cooperation on other regular network structures. The figure complements Figs. 2 and S1 by adding degree-4 (left panels) and degree-8 (right panels) ring lattices and the complete network, starting from 1% and 50% of initial C's. Rings behave similarly to square lattices, better supporting the invasion of C's (slightly lower $r_{\rm inv}$ for 1% initial C's). Typically, in ring lattices, the D's at the boundary of a small C-cluster (e.g., those next to a C in the main loop) have less D-neighbors than boundary D's in square lattices, so the that D-to-C strategy changes requires a lower return at low levels of C. The increased number of simulations not ended in all-C or all-D w.r.t. square lattices (dots in the open interval $(0,1)$, especially for degree-4 and 1% initial C's) is due to the slower convergence, because of the essentially one-dimensional ring structure. The all-to-all connection is the most demanding structure for the invasion of C's (note the different scale of the r-axis in panel **e**), and even for its fixation starting from 50% initial C's (compare with Fig. S1). The complete network is in a stalemate starting from 50% initial C's with intermediate returns. Stalemate at the initial state is possible on any network structure (see, e.g., the simple network of Fig. 1**b** analyzed in Sect. S10), though we observed it in our numerical simulations only for the complete network. It typically requires significant C-levels, since isolated C's are willing to change, though we observed it also at 1% initial C's for r slightly larger than $r_{\rm inv}$. (See Sect. S12 for more details on the numerical results.)

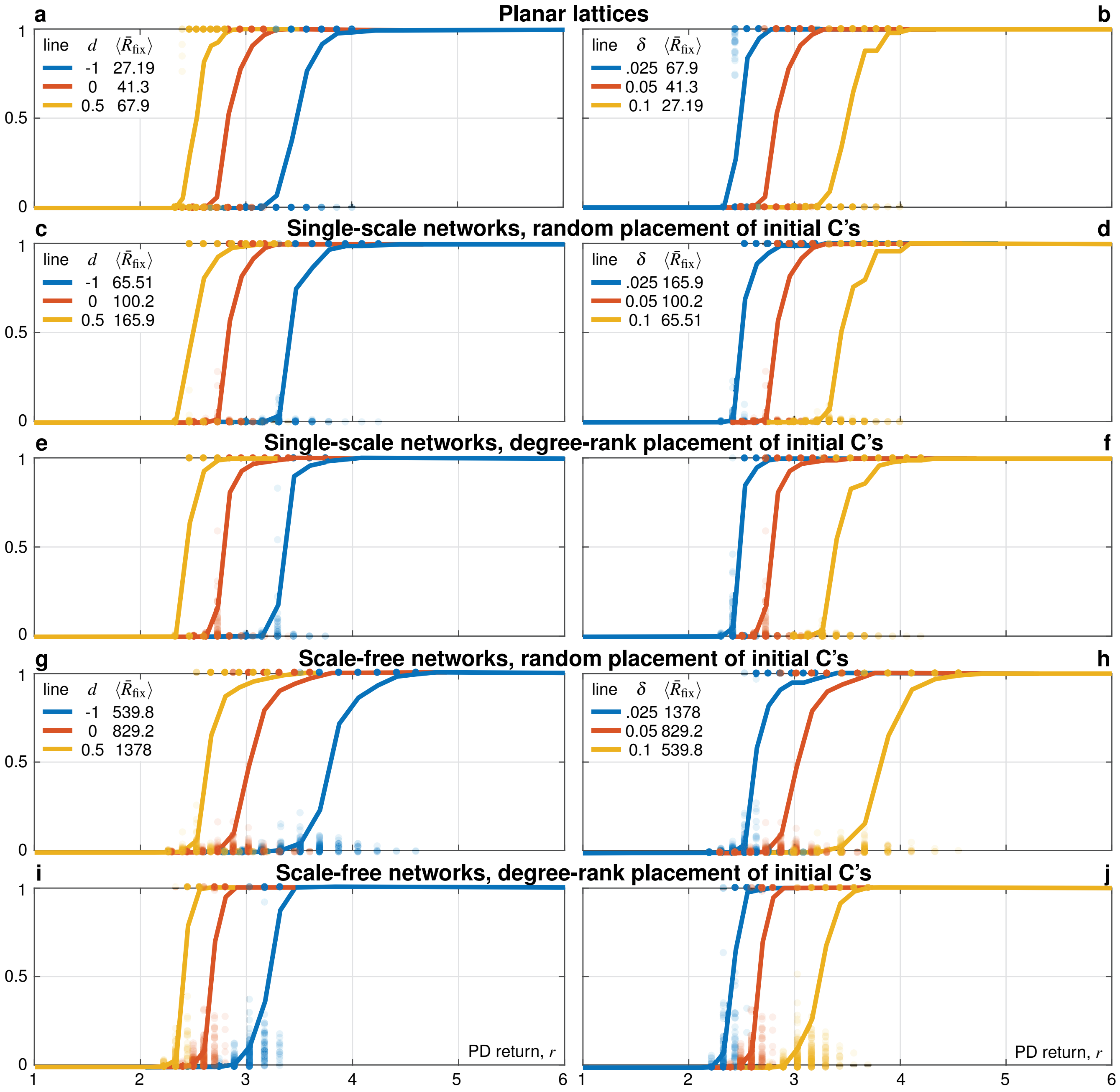

Figure S3. Sensitivity analysis. The figure complements Fig. 2 by reproducing the simulations with average degree $\langle k \rangle = 4$ and predictive horizon $h = 3$ (red in the left panels of Fig. 2) for two different (larger/smaller) values of the reciprocity parameter d (0.5 and -1, halving/doubling the reciprocity-biased rate of strategy update δ_d; left column) and of the rate of strategy update δ (0.1 and 0.025, double/half of the reference value; right column). As expected at points (v) and (viii) of the Analytical results, super/sub-normal reciprocity (d positive/negative) as well as higher/lower inertia to change (lower/higher δ) result in lower/higher thresholds r_{inv} and r_{fix}.

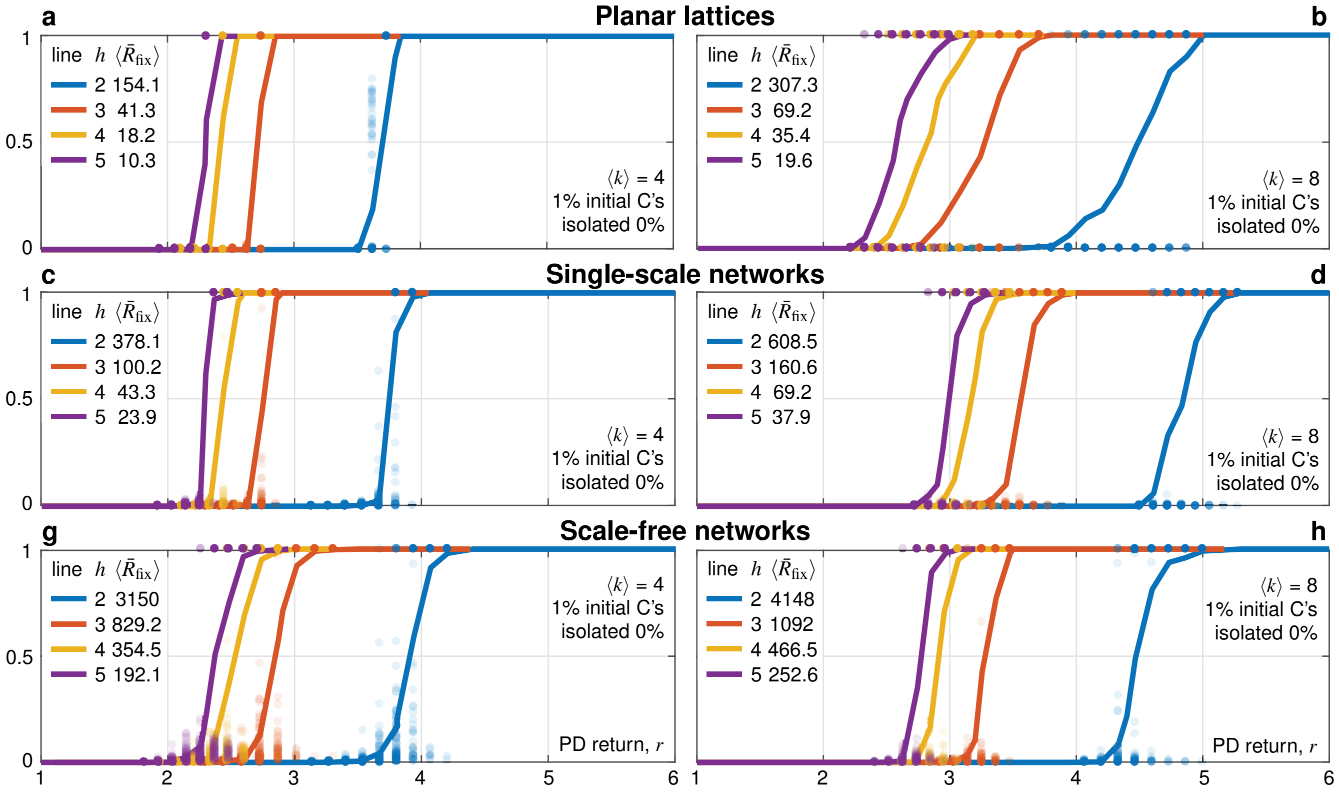

Figure S4. Invasion, persistence, and fixation of cooperation under random pair placement of the initial C's. The figure complements Fig. 2 by reproducing the simulations with random placement of the initial C's by randomly selecting pairs of C-neighbors instead of single agents. The 10 initial C's (1% of $N = 1000$ nodes) are iteratively selected as follows: each time a selected node has no C-neighbors, the next node is selected among its neighbors; the last node is either selected to pair the previous one, or among the D's connected to C's. Both r_{inv} and r_{fix}, as well as their gap, get reduced w.r.t. random placement in Fig. 2 (same panel labels). Recall that isolated C's switch to D at first strategy revision, so that starting with all isolated C's it is always possible to end in the trivial stalemate all-D. Note that degree-rank-pair-placement—selecting initial C's according to degree-ranking by pairing isolated ones—would have reproduced the results of Fig. 2, as random and degree-rank selections are essentially equivalent for single-scale networks and degree-rank-C-placement is unlikely to leave isolated C's in scale-free networks.

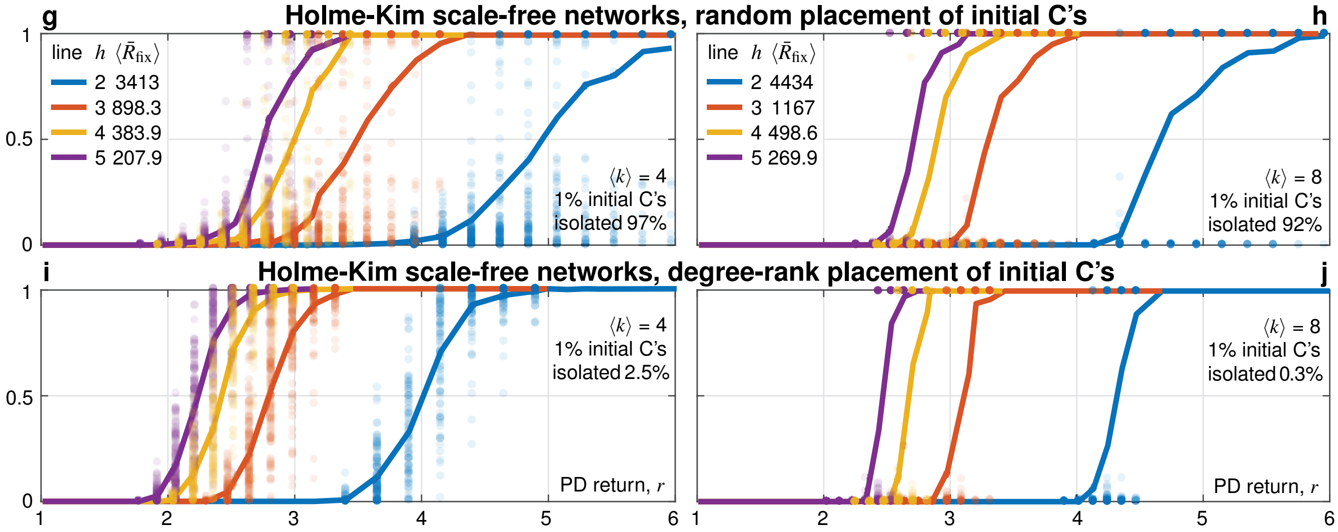

Figure S5. Invasion, persistence, and fixation of cooperation in scale-free networks with medium-high transitivity. Network transitivity is the average probability that the neighbors of a node are neighbors themselves (it is best known as network clustering; we use transitivity to avoid confusion with C-clusters, i.e., maximal groups of connected C's). We checked the robustness of our results with respect to a different type of scale-free network (Holme-Kim model,[84] HK) with tunable transitivity, since this network feature has been shown to favor cooperation under imitative update,[30,34] although it vanishes with the size of Barabási-Albert networks.[83] (see Sect. S11 for details on the BA and HK algorithms). The resulting transitivity (average over 100 networks) is 0.74 for average degree $\langle k \rangle = 4$ (left) and 0.28 for $\langle k \rangle = 8$ (right) (respectively 0.027 and 0.037 in Fig. 2; transitivity is known to increase/decrease with $\langle k \rangle$ in BA/HK networks[83,84]). Although the theoretical degree distribution for large size is the same of a BA network, the effect of raising transitivity in a finite network is an increase of nodes with low and high degree to the detriment of nodes with intermediate degree (checked on average on our 100 networks). This 'finite size effect' explains our results: slightly lower r_{inv} and higher r_{fix} compared with the same panel in Fig. 2. Invasion is indeed favored by the enhanced initial presence of low-connected D's, who change to C under mild returns if connected to a C, whereas fixation is hindered by the enhanced initial presence of D-hubs. The increased number of simulations not ended in all-C or all-D (dots in the open interval $(0,1)$, essentially in the left panels where transitivity is very high, w.r.t. Fig. 2) can be also explained in terms of the loss of nodes with intermediate degree. On one hand, the loss makes the evolution slower, possibly forming bottlenecks through which cooperation must percolate, so that some of the dots at significant C-levels might denote simulations ending in all-C on a longer timescale. On the other hand, the loss makes nontrivial stalemates and fluctuations more frequent, as D-nodes with high degree can prevent the convergence to all-C even under degree-rank-C-placement. (See Sect. S12 for more details on the numerical results.)

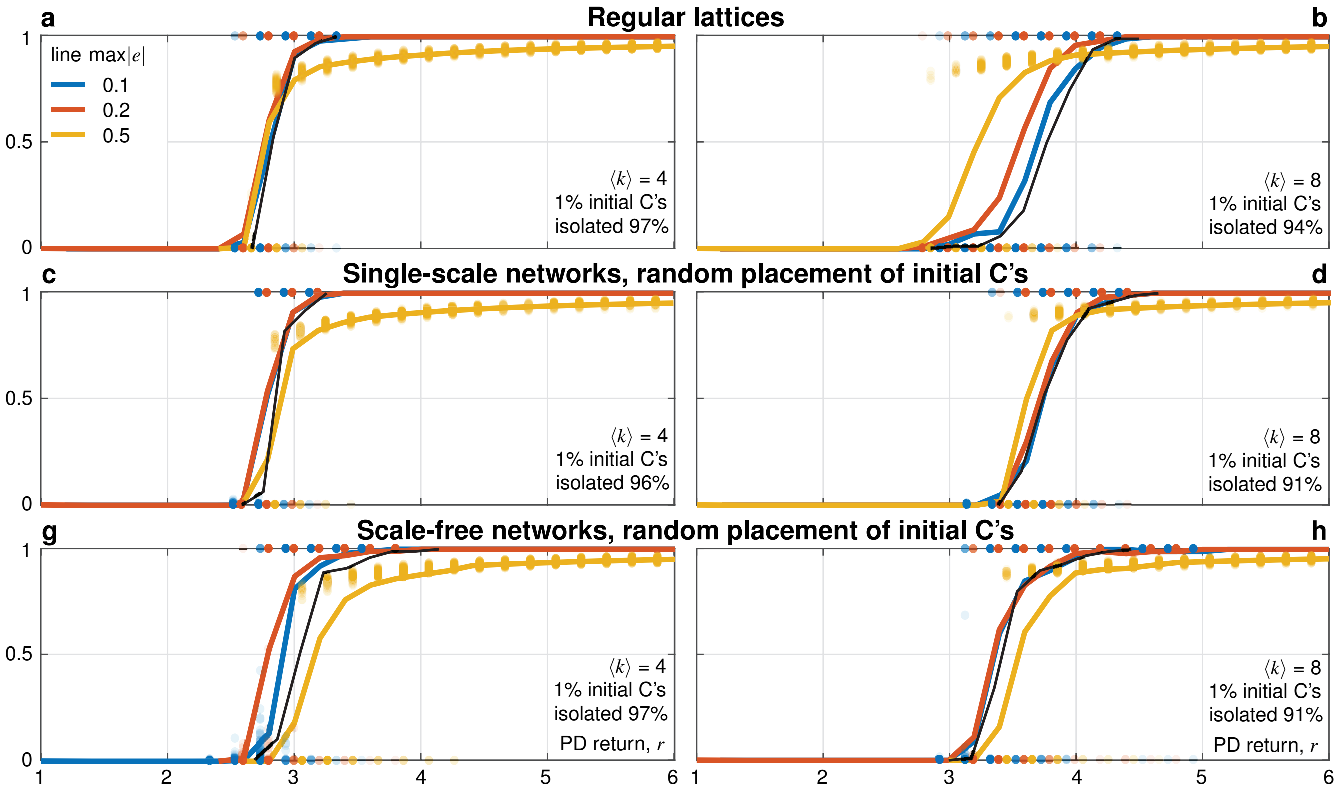

Figure S6. Robustness analysis w.r.t. computational errors. The figure complements Fig. 2 by reproducing the simulations with predictive horizon $h = 3$ and random placement of the initial C's (red in panels **a**–**d**, **g**, **h** of Fig. 2; thin black here with same panel labels) for different relative errors in computing model-predictions. When C and D revising agents compute their expected C-payoff π^h_{CC} and π^h_{DC} and their expected D-payoff π^h_{CD} and π^h_{DD}, respectively, a 10-20-50% random error is added (e.g., π^h_{CC} is replaced with $\pi^h_{\mathrm{CC}}(1+e)$ with e uniformly drawn in $[-0.1, 0.1]$ for 10%) before the expected gains $\Delta\pi^h_{\mathrm{C}} = \pi^h_{\mathrm{CD}} - \pi^h_{\mathrm{CC}}$ and $\Delta\pi^h_{\mathrm{D}} = \pi^h_{\mathrm{DC}} - \pi^h_{\mathrm{DD}}$ are computed. All panels show that the results of Fig. 2 are very robust to computational errors, including network reciprocity (compare left vs right panels). The reason is that, even with significant relative errors, the sign of the expected payoff gains $\Delta\pi^h_{\mathrm{C}}$ and $\Delta\pi^h_{\mathrm{D}}$ is often unchanged w.r.t. the same simulation in Fig. 2 (with same random drawing for abstention and strategy update). The two general effects are: lowering the invasion threshold r_{inv}, because D-agents connected to an initial C can change to C by error and trigger invasion even when the correct expected gain is negative; and raising the fixation threshold r_{fix}, especially for considerable error rates, because larger expected gains are needed to stabilize the state all-C.

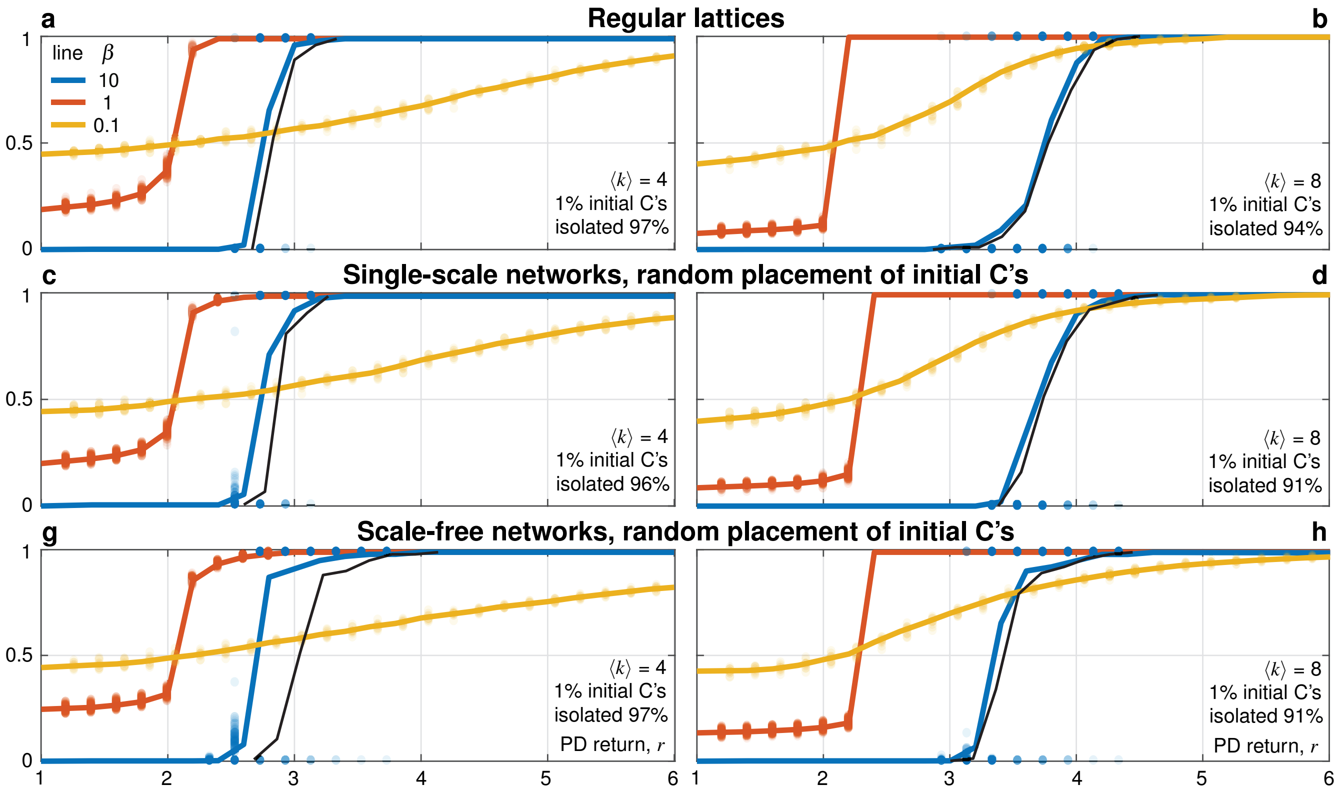

Figure S7. Robustness analysis w.r.t. irrational behaviors. The figure complements Fig. 2 by reproducing the simulations with predictive horizon $h = 3$ and random placement of the initial C's (red in panels **a**–**d**, **g**, **h**; thin black here with same panel labels) for different degrees of agents' irrationality. When C and D agents revise their strategies, instead of changing strategy if, and only if, their expected payoff gains $\Delta\pi_C^h$ and $\Delta\pi_D^h$ are positive, they change with the probability given by the Fermi exponential function $\exp(\beta\Delta\pi)/(1+\exp(\beta\Delta\pi))$, where parameter β (uniform across the population) controls their degree of rationality (Fig. 2 corresponds to the limit $\beta \to \infty$ of full-rationality). All panels show that the results of Fig. 2 are robust to significant degrees of irrationality (with $\beta = 1$, the probability to change strategy under an expected payoff gain, resp. loss, of one unit is 0.73, resp. 0.27). Note, however, that with $\beta = 0.1$ (52.5% and 47.5% strategy change under unitary expected gain and loss) network reciprocity is no longer present, suggesting that higher connectivity helps cooperation under a totally random update. Even more significantly than computational errors (see Fig. S6), the main effect of irrational decisions is to lower the invasion threshold r_{inv}, because small clusters of C's can form by error, and the C's in a cluster remain C under a milder requirement on the game return r, than if isolated. At the same time, their D-neighbors more likely change to C. The effect of raising the fixation threshold r_{fix} is visible only at large degrees of irrationality ($\beta = 0.1$).

Supplementary Methods

S1 The probability to play p_t

After $t-1 \geq 0$ consecutive abstentions following an exploitation by the neighbor j, the probability p_{ij} that the C-agent i agrees to play with j at the next game round is set to $p_t = 1-(1-\delta_d)^t$. It is the probability that j has revised her strategy at least once ever since the exploitation, according to the reciprocity-biased rate of strategy update δ_d. Recall that $\delta_d = (1-d)\delta \in (0,1)$ is equal to, resp. smaller/larger than, the actual update rate δ for for normally ($d=0$), resp. super/sub- (d positive/negative), reciprocating agents.

If the (i,j) PD interaction takes place at the next round, then i sets p_{ij} to 1 or to $p_1 = \delta_d$ depending on whether j cooperates or defects. Otherwise, i updates p_{ij} to p_{t+1}. Note that p_{t+1} can be obtained with the recursion

$$p_{t+1} = 1-(1-p_t)(1-\delta_d) = (1-\delta_d)p_t + \delta_d, \quad t \geq 1, \tag{S1}$$

where $(1-p_t)(1-\delta_d)$ is the probability that j did not revise strategy after the last $t+1$ rounds, the one in which j exploited i and the following t rounds skipped by i. We set $p_0 = 1$ and, whenever needed in the following, t_{ij} denotes the integer giving $p_{ij} = p_{t_{ij}}$ for the neighbor pair (i,j).

S2 The probability of getting exploited P^t_{CD}

When the C-agent i revises her strategy, she assumes the neighbor j to be a D, and to play as such in all h rounds of the predictive horizon, if and only if $p_{ij} < 1$ (even though j might have changed to C since j last exploited i). Let $p_{ij} = p_{t_{ij}} < 1$ for some $t_{ij} \geq 1$ and $P^t_{\mathrm{CD}}(t_{ij})$ be the probability that i expects to have, behaving as C, to play with j at round t of the predictive horizon. Obviously, $P^1_{\mathrm{CD}}(t_{ij}) = p_{t_{ij}}$ (the t_{ij}-argument, often omitted in the following, makes initialization explicit). For $t > 1$, $P^t_{\mathrm{CD}}(t_{ij})$ is computed by means of the following recursion:

$$P^{t+1}_{\mathrm{CD}} = P^t_{\mathrm{CD}}\delta_d + (1-P^t_{\mathrm{CD}})\big((1-\delta_d)P^t_{\mathrm{CD}} + \delta_d\big) = \delta_d + (1-\delta_d)P^t_{\mathrm{CD}}(1-P^t_{\mathrm{CD}}). \tag{S2}$$

That is, if i is exploited by j at round t, she will then play at round $t+1$ with probability $p_1 = \delta_d$ (first term after the first equal sign in (S2)); otherwise P^t_{CD} is updated as p_t in (S1) (second term).

The dynamics of P^t_{CD} is analyzed in Fig. S8. As $t \to \infty$, P^t_{CD} converges (independently of the initialization) to the infinite-horizon limit used in condition (1) and in the fixation threshold (2) in the main text, i.e.,

$$P^\infty_{\mathrm{CD}} = \frac{1}{2}\frac{\sqrt{4\delta_d - 3\delta_d^2} - \delta_d}{1-\delta_d} \simeq \sqrt{\delta_d} \ \text{ for small } \delta_d. \tag{S3}$$

The limit is reached monotonically if $\delta_d < 1/3$ (from below if $P^1_{\mathrm{CD}} < P^\infty_{\mathrm{CD}}$ or $P^1_{\mathrm{CD}} > 1-P^\infty_{\mathrm{CD}}$; from above if $P^\infty_{\mathrm{CD}} < P^1_{\mathrm{CD}} \leq 1-P^\infty_{\mathrm{CD}}$). This condition is met in the numerical analysis, where the largest δ_d is 0.1, corresponding to the perturbed value used in Fig. S3 with respect to the reference value $\delta_d = 0.05$ (see Table 1 in the main text).

Starting with $p_{ij} = \delta_d$ (i.e., $t_{ij} = 1$), the probabilities p_t and P^t_{CD} are listed in Table S1 for $t \geq 1$ (first and second columns). Both probabilities have linear (1-st-order) leading-term in δ_d, with same coefficient equal to t. Moreover, from the right-most side of (S2), it follows that the coefficient of the leading-term in P^{t+1}_{CD} is 1 plus the coefficient in P^{t+1}_{CD} (up to 1-st-order in δ_d, the right-most side of (S2) is $\delta_d + P^t_{\mathrm{CD}}$), so that $P^t_{\mathrm{CD}}(t_{ij}) \simeq (t_{ij}+t-1)\delta_d$ for small δ_d.

Note that $P^t_{\mathrm{CD}}(1)$ is also used by the revising D-agent i to evaluate the chances to be exploited, if changing to C, by her D-neighbors. Similarly, $P^t_{\mathrm{CD}}(t_{ji})$ is used by the revising C or D agent i to evaluate the chances to exploit, behaving as D, the C-neighbor j with $p_{ji} = p_{t_{ji}}$, $t_{ji} \geq 0$.

S3 The probability to reciprocate P^t_{CC}

When the C-agent i revises her strategy, she assumes the neighbor j to be a C, and to play as such in all h rounds of the predictive horizon, if and only if $p_{ij} = 1$ (j was indeed a C in the last round, but she might be revising as well). Let $p_{ij} = 1$, $p_{ji} = p_{t_{ji}}$ for some $t_{ji} \geq 0$, and $P^t_{\mathrm{CC}}(t_{ji})$ be the probability that i expects to have to play with j at round t of the predictive horizon. If $t_{ji} = 0$, then i and j reciprocated cooperation in the last round, so that $P^t_{\mathrm{CC}}(0) = 1$ for all $t \geq 1$. Otherwise, j stopped playing after getting exploited by i, who later turned C. In both cases we have $P^1_{\mathrm{CC}}(t_{ji}) = p_{t_{ji}}$, while for $t > 1$, $P^t_{\mathrm{CC}}(t_{ji})$ is computed by means of the following recursion:

$$P^{t+1}_{\mathrm{CC}}(t_{ji}) = P^t_{\mathrm{CC}}(t_{ji}) + (1-P^t_{\mathrm{CC}}(t_{ji}))p_{t_{ji}+t} = 1-(1-\delta_d)^{t_{ji}+t} + P^t_{\mathrm{CC}}(t_{ji})(1-\delta_d)^{t_{ji}+t}. \tag{S4}$$

That is, if j does play at round t, she will certainly play at round $t+1$ (first term after the first equal sign in (S4)); otherwise she will not be playing since round 1 and she will therefore play at round $t+1$ with probability $p_{t_{ji}+t}$ (second term).

The probability $P^t_{\mathrm{CC}}(t_{ji})$ monotonically increases to 1 (independently of t_{ji}). Starting with $p_{ji} = \delta_d$ (i.e., $t_{ji} = 1$), the probability P^t_{CC} is listed in Table S1 for $t \geq 1$ (third column). The leading δ_d-term is linear (1-st-order) and its coefficient in

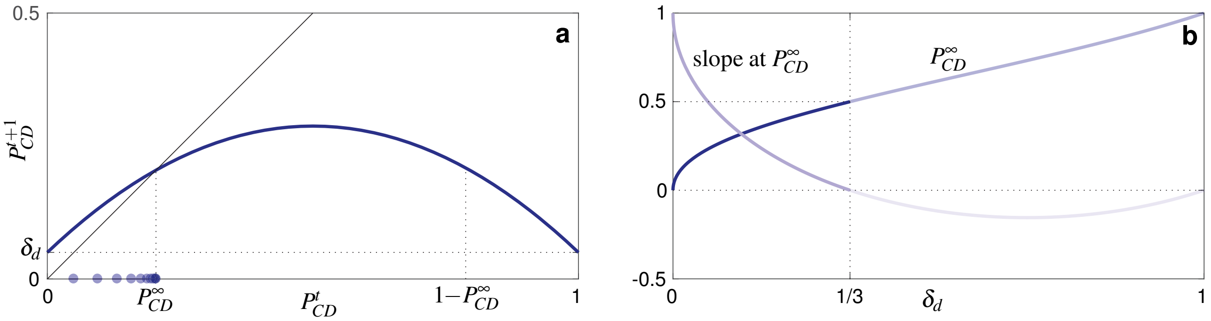

Figure S8. The dynamics of the recursion (S2). (**a**) The right-hand side of (S2) for $\delta_d = 0.05$ (solid thick line; the thin line is the diagonal) and the probabilities $P^t_{\mathrm{CD}}(1), t = 1, \ldots, 10$ (dots). (**b**) The fixed point P^{∞}_{CD} and the asymptotic rate of convergence (the local slope $1-\sqrt{\delta_d(4-3\delta_d)}$ of the right-hand side of (S2) at the fixed point) as a function of δ_d. The slope is in $(-1, 1)$ (asymptotic stability) for all $\delta_d \in (0, 1)$ and in $(0, 1)$ (monotonic convergence) for $\delta_d \in (0, 1/3)$.

t	p_t	P^t_{CD}	P^t_{CC}
1	δ_d	δ_d	δ_d
2	$2\delta_d - \delta_d^2$	$2\delta_d - 2\delta_d^2 + \delta_d^3$	$3\delta_d - 3\delta_d^2 + \delta_d^3$
3	$3\delta_d - 3\delta_d^2 + \delta_d^3$	$3\delta_d - 8\delta_d^2 + 15\delta_d^3 - 17\delta_d^4 + 12\delta_d^5 \cdots + \delta_d^7$	$6\delta_d - 15\delta_d^2 + 20\delta_d^3 - 15\delta_d^4 + 6\delta_d^5 - \delta_d^6$
4	$4\delta_d - 6\delta_d^2 + 4\delta_d^3 - \delta_d^4$	$4\delta_d - 20\delta_d^2 + 80\delta_d^3 - 234\delta_d^4 + 525\delta_d^5 \cdots + \delta_d^{15}$	$10\delta_d - 45\delta_d^2 + 120\delta_d^3 - 210\delta_d^4 + 252\delta_d^5 \cdots - \delta_d^{10}$
5	$5\delta_d - 10\delta_d^2 + 10\delta_d^3 - 5\delta_d^4 + \delta_d^5$	$5\delta_d - 40\delta_d^2 + 276\delta_d^3 - 1514\delta_d^4 + 6871\delta_d^5 \cdots + \delta_d^{31}$	$15\delta_d - 105\delta_d^2 + 455\delta_d^3 - 1365\delta_d^4 + 3003\delta_d^5 \cdots + \delta_d^{15}$

Table S1. Probabilities p_t, $P^t_{\mathrm{CD}}(1)$, and $P^t_{\mathrm{CC}}(1)$ for $t = 1, \ldots, 5$.

$P^{t+1}_{\mathrm{CC}}(t_{ji})$ is $t_{ji} + t$ plus the coefficient in $P^t_{\mathrm{CC}}(t_{ji})$ (up to 1-st-order in δ_d, the right-most side of (S4) is $(t_{ji} + t)\delta_d + P^t_{\mathrm{CC}}$). This results in $P^t_{\mathrm{CC}}(t_{ji}) \simeq ((2t_{ij} - 1)t + t^2)/2\delta_d$ for small δ_d. For sufficiently small δ_d, we hence have

$$P^t_{\mathrm{CC}}(\tau) > P^t_{\mathrm{CD}}(\tau) \ \text{ for any integer } \tau \geq 0 \text{ and } t > 1. \tag{S5}$$

We now show that inequality (S5) holds true for any $\delta_d \in (0, 1)$ (recall that $\delta_d \in (0, 1)$ means that there is direct reciprocity in the C-strategy). From the first right-hand side in (S4), we see that $P^{t+1}_{\mathrm{CC}}(\tau)$ is a convex combination (with positive $P^t_{\mathrm{CC}}(\tau)$) of 1 and $p_{\tau+t} < 1$, so that $P^t_{\mathrm{CC}}(\tau) > p_{\tau+t-1}$ for any $t > 1$. Similarly, from the first right-hand side in (S2), we see that $P^{t+1}_{\mathrm{CD}}(\tau)$ is a convex combination (with positive $P^t_{\mathrm{CD}}(\tau)$) of δ_d, the smallest of the p_t's, and the transformation of $P^t_{\mathrm{CD}}(\tau)$ by rule (S1). By construction, we hence have $P^t_{\mathrm{CD}}(\tau) < p_{\tau+t-1}$ for any $t > 1$. The surplus of $P^h_{\mathrm{CC}}(\tau)$ over $P^h_{\mathrm{CD}}(\tau)$ is graphed in Fig. S9.

Note that $P^t_{\mathrm{CC}}(t_{ji})$ is also used by the revising D-agent i to evaluate the chances to cooperate, if changing to C, with her C-neighbors.

S4 The expected payoff gain $\Delta\pi^h_{\mathrm{C}}$

The revising C-agent i computes her expected payoffs collected in the next h rounds behaving as D, π^h_{CD}, or as C, π^h_{CC}, as follows:

- Initialize $\pi^h_{\mathrm{CD}} = 0$ and $\pi^h_{\mathrm{CC}} = 0$ and consider the sums

$$S^h_{\mathrm{CD}}(\tau) = \sum_{t=1}^{h} P^t_{\mathrm{CD}}(\tau) \quad \text{and} \quad S^h_{\mathrm{CC}}(\tau) = \sum_{t=1}^{h} P^t_{\mathrm{CC}}(\tau). \tag{S6}$$

- For each $p_{ij} = 1$, add $rS^h_{\mathrm{CD}}(t_{ji})$ to π^h_{CD}; these are the payoffs expected by exploiting C-neighbors.
- For each $p_{ij} = 1$, add $(r-1)S^h_{\mathrm{CC}}(t_{ji})$ to π^h_{CC}; these are the payoffs expected by cooperating with C-neighbors.
- For each $p_{ij} < 1$, subtract $S^h_{\mathrm{CD}}(t_{ij})$ from π^h_{CC}; these are the losses expected for being exploited by D-neighbors.

Then, $\Delta\pi^h_{\mathrm{C}} = \pi^h_{\mathrm{CD}} - \pi^h_{\mathrm{CC}}$, i.e.,

$$\begin{aligned} \Delta\pi^h_{\mathrm{C}} &= \sum_{p_{ij}=1} \left(rS^h_{\mathrm{CD}}(t_{ji}) - (r-1)S^h_{\mathrm{CC}}(t_{ji})\right) + \sum_{p_{ij}<1} S^h_{\mathrm{CD}}(t_{ij}) \\ &= -r \sum_{p_{ij}=1} \underbrace{\left(S^h_{\mathrm{CC}}(t_{ji}) - S^h_{\mathrm{CD}}(t_{ji})\right)}_{>0 \text{ for } h \geq 2} + \sum_{p_{ij}=1} S^h_{\mathrm{CC}}(t_{ji}) + \sum_{p_{ij}<1} S^h_{\mathrm{CD}}(t_{ij}). \end{aligned} \tag{S7}$$

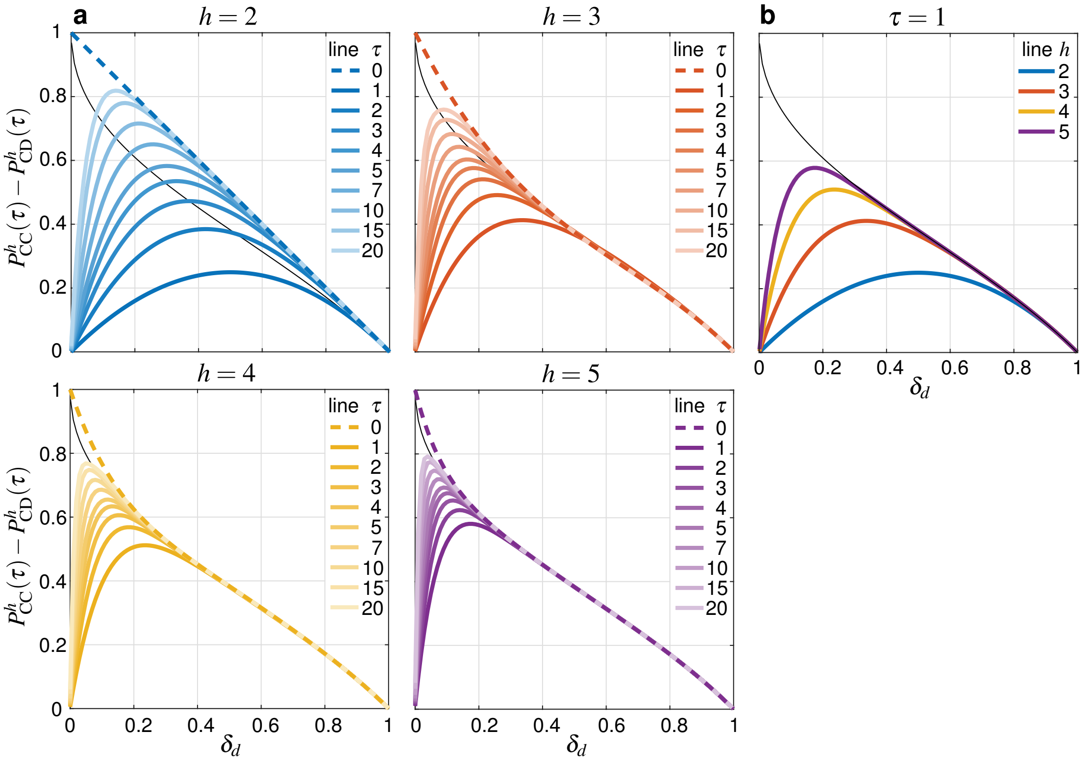

Figure S9. Surplus of the probability to reciprocate $P^h_{CC}(\tau)$ over the probability of getting exploited $P^h_{CD}(\tau)$. Plot of the surplus versus the reciprocity-biased rate of strategy update δ_d. (**a**) Different values of the predictive horizon ($h = 2, \ldots, 5$) and initializations of the probability to play at the next round ($\tau = 0, \ldots, 9$). (**b**) The case $\tau = 1$ in a single panel. Colored lines: $P^h_{CC}(\tau) - P^h_{CD}(\tau)$; black thin line: $1 - P^\infty_{CD}$ for comparison.

Note that, by inequality (S5), the elements in the first sum in the right-most side of (S7) are all positive if $h \geq 2$ (with $\delta_d \in (0,1)$); for $h = 1$ they are all zero. Thus, provided agent i expects to have some C-neighbors (so that the sum is not void), the multi-step prediction ($h \geq 2$) and direct reciprocity in the C-strategy ($\delta_d \in (0,1)$) make the expected payoff gain $\Delta\pi^h_C$ negative for sufficiently large values of the PD return r. The condition on r for i to remain C is

$$r > \frac{\sum_{p_{ij}=1} S^h_{CC}(t_{ji}) + \sum_{p_{ij}<1} S^h_{CD}(t_{ij})}{\sum_{p_{ij}=1} \left(S^h_{CC}(t_{ji}) - S^h_{CD}(t_{ji})\right)} = 1 + \frac{\sum_{p_{ij}=1} S^h_{CD}(t_{ji}) + \sum_{p_{ij}<1} S^h_{CD}(t_{ij})}{\sum_{p_{ij}=1} \left(S^h_{CC}(t_{ji}) - S^h_{CD}(t_{ji})\right)}. \tag{S8}$$

For $h = 1$, we have

$$\Delta\pi^1_C = \sum_{p_{ij}=1} P^1_{CC}(t_{ji}) + \sum_{p_{ij}<1} P^1_{CD}(t_{ij}) = \sum_{p_{ij}=1} p_{ji} + \sum_{p_{ij}<1} p_{ij} > 0, \tag{S9}$$

i.e., revising C-agents change to D, irrespectively of r.

S5 The expected payoff gain $\Delta\pi^h_D$

The revising D-agent i computes her expected payoffs collected in the next h rounds behaving as C, π^h_{DC}, or as D, π^h_{DD}, as follows:

- Initialize $\pi^h_{DC} = 0$ and $\pi^h_{DD} = 0$ and consider the sums S^h_{CD} and S^h_{CC} in (S6).
- For each $p_{ji} < 1$, add $(r-1)S^h_{CC}(t_{ji})$ to π^h_{DC}; these are the payoffs expected by cooperating with C-neighbors.
- For each $p_{ji} < 1$, add $rS^h_{CD}(t_{ji})$ to π^h_{DD}; these are the payoffs expected by exploiting C-neighbors.
- For each $p_{ji} = 1$, subtract $S^h_{CD}(1)$ from π^h_{DC}; these are the losses expected for being exploited by D-neighbors.

Then, $\Delta\pi_{\rm D}^h = \pi_{\rm DC}^h - \pi_{\rm DD}^h$, i.e.,

$$\begin{aligned}\Delta\pi_{\rm D}^h &= \sum_{p_{ji}<1} \left((r-1)S_{\rm CC}^h(t_{ji}) - rS_{\rm CD}^h(t_{ji})\right) - \sum_{p_{ji}=1} S_{\rm CD}^h(1) \\ &= r\sum_{p_{ji}<1} \underbrace{\left(S_{\rm CC}^h(t_{ji}) - S_{\rm CD}^h(t_{ji})\right)}_{>0 \text{ for } h\geq 2} - \sum_{p_{ji}<1} S_{\rm CC}^h(t_{ji}) - \sum_{p_{ji}=1} S_{\rm CD}^h(1). \end{aligned} \tag{S10}$$

By the same arguments discussed in the previous section, one C-neighbor is enough for the multi-step prediction ($h \geq 2$) and direct reciprocity ($\delta_d \in (0,1)$) to make the expected payoff gain $\Delta\pi_{\rm D}^h$ positive for sufficiently large values of the PD return r. The condition on r for i to change to C is

$$r > \frac{\sum_{p_{ji}<1} S_{\rm CC}^h(t_{ji}) + \sum_{p_{ji}=1} S_{\rm CD}^h(1)}{\sum_{p_{ji}<1}\left(S_{\rm CC}^h(t_{ji}) - S_{\rm CD}^h(t_{ji})\right)} = 1 + \frac{\sum_{p_{ji}<1} S_{\rm CD}^h(t_{ji}) + \sum_{p_{ji}=1} S_{\rm CD}^h(1)}{\sum_{p_{ji}<1}\left(S_{\rm CC}^h(t_{ji}) - S_{\rm CD}^h(t_{ji})\right)}. \tag{S11}$$

For $h=1$, we have

$$\Delta\pi_{\rm D}^1 = -\sum_{p_{ji}<1} P_{\rm CC}^1(t_{ji}) - \sum_{p_{ji}=1} P_{\rm CD}^1(1) = -\sum_{p_{ji}<1} p_{ji} - \sum_{p_{ji}=1} p_1 < 0, \tag{S12}$$

i.e., revising D-agents remain D, irrespectively of r.

Also note that condition (S11) is typically more demanding than (S8), on equal neighborhoods (same number of p_{ji}'s <1 in (S11) and of p_{ij}'s $=1$ in (S8)). Indeed, each (positive) element of the sum in the common denominator grows with $t_{ji} \geq 1$ (and is maximal for $t_{ji}=0$), as each element of the sum $S_{\rm CC}^h(t_{ji}) - S_{\rm CD}^h(t_{ji}) = \sum_{t=1}^h \left(P_{\rm CC}^t(t_{ji}) - P_{\rm CD}^t(t_{ji})\right)$ does so (checked numerically, see Fig. S9**a**), and the t_{ji}'s are expected to be higher (or zero) for a C-agent i.

S6 The infinite-horizon limit

With an infinite predictive horizon ($h \to \infty$), the sums $S_{\rm CD}^h(\tau)$ and $S_{\rm CC}^h(\tau)$ (defined in (S6)) diverge with $S_{\rm CD}^h/h \to P_{\rm CD}^\infty$ and $S_{\rm CC}^h/h \to 1$ independently of τ. Consequently, for a C and a D agent with degree k and $k_{\rm C} \leq k$ C-neighbors, the expected payoff gains $\Delta\pi_{\rm C}^h$ and $\Delta\pi_{\rm D}^h$ (from eqs. (S7) and (S10)) are unbounded with

$$\Delta\pi_{\rm C}^h/h \to -rk'_{\rm C}(1-P_{\rm CD}^\infty) + k'_{\rm C} + (k-k'_{\rm C})P_{\rm CD}^\infty, \tag{S13a}$$

$$\Delta\pi_{\rm D}^h/h \to rk_{\rm C}(1-P_{\rm CD}^\infty) - k_{\rm C} - (k-k_{\rm C})P_{\rm CD}^\infty, \tag{S13b}$$

where, for a C, $k'_{\rm C} \leq k_{\rm C}$ is the number of $p_{ij}=1$. Solving $\Delta\pi_{\rm C}^h/h < 0$ and $\Delta\pi_{\rm D}^h/h > 0$ in the limits in (S13) gives condition (1) in the main text.

S7 The fixation threshold $R_{\rm fix}$

From the conditions (S8) and (S11) it is clear that, under the multi-step prediction ($h \geq 2$) and direct reciprocity ($\delta_d \in (0,1)$), there is a threshold $R_{\rm fix}$ on r above which $\Delta\pi_{\rm C}^h < 0$ and $\Delta\pi_{\rm D}^h > 0$ for any C and D agent i with at least one expected C-neighbor (i.e., $p_{ij}=1$ if i is a C, $p_{ji}<1$ if i is a D, for at least one j). If the PD return is above threshold, cooperation fixates in any network structure starting from any pair of connected C's. The threshold $R_{\rm fix}$ of course depends on the network structure. It is the maximal value attained by the right-hand sides in (S8) and (S11) over all possible choices of the node i and over all possible configurations of its neighborhood, restricting the search to configurations with at least one expected C-neighbor that can be reached from an initial state (a state with $p_{ij}=p_{ji}=1$ for all connected pairs (i,j) and $p_{ij}=p_{ji}=0$ otherwise).

In the following we derive an upper bound to $R_{\rm fix}$ consistent with the infinite-horizon limit $R_{\rm fix}^\infty$ in eq. (2) (main text). The denominators in (S8) and (S11) are minimized if the sum comprises a single element, i.e., if agent i expects to have only one C-neighbor, say j. Moreover, as discussed at the end of Sect. S5 (and visualized in Fig. S9**a**), the single j-element grows with $t_{ji} \geq 1$ (and is maximal for $t_{ji}=0$), so that the denominators in (S8) and (S11) are the smallest for $t_{ji}=1$. At the numerators of the right-most sides of (S8) and (S11) there are k_i terms of the kind $S_{\rm CD}^h(\tau)$, where k_i is the degree of node i. The value of $\tau \geq 0$ that maximizes $S_{\rm CD}^h(\tau)$ unfortunately depends on both h and δ_d, so we cannot upper bound the numerator by a specific choice of τ. However, we note that $S_{\rm CD}^h(\tau)$ is upper bounded by $1+(h-1)P_{\rm CD}^\infty$ (checked numerically, see Fig. S10).

The fixation threshold $R_{\rm fix}$ is hence upper bounded by

$$R_{\rm fix} < \bar{R}_{\rm fix} = 1 + k_{\max}\frac{1+(h-1)P_{\rm CD}^\infty}{S_{\rm CC}^h(1) - S_{\rm CD}^h(1)}, \tag{S14}$$

obtained by taking i as the node with maximal degree $k_{\max}$. The denominator $S_{\rm CC}^h(1) - S_{\rm CD}^h(1)$ can be easily tabulated for different h from Table S1. The leading δ_d-term is linear (1-st-order) and its coefficient is given by the sum of the leading coefficients in $P_{\rm CC}^t(1)$, t from 1 to h (third column in Table S1) minus the sum of the leading coefficients in $P_{\rm CD}^t(1)$ (second column), resulting in $S_{\rm CC}^h(1) - S_{\rm CD}^h(1) \simeq h(h^2-1)/6\delta_d$ for small δ_d.

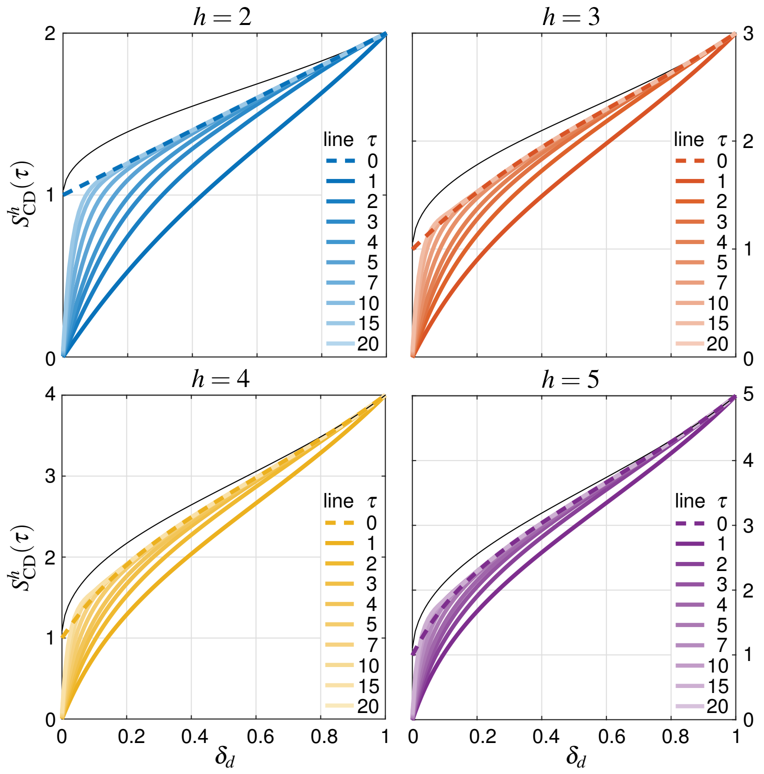

Figure S10. The sum $S^h_{\mathrm{CD}}(\tau)$. Plot of the sum versus the reciprocity-biased rate of strategy update δ_d for different values of the predictive horizon ($h = 2,\ldots,5$) and initializations of the probability to play at the next round ($\tau = 0,\ldots,9$). Colored lines: $S^h_{\mathrm{CD}}(\tau)$; black thin line: the upper bound $1+(h-1)P^\infty_{\mathrm{CD}}$.

The bound $\bar{R}_{\mathrm{fix}}$ is very conservative, especially for small δ_d, as confirmed by all our simulations. The reason is that the minimization of the denominators in (S8) or in (S11) and the maximization of the numerators of the right-most sides do not concomitantly occur. For example, the denominators vanish with δ_d if the t_{ji}'s being all ≥ 1 (p_{ji}'s < 1), but in this situation the numerators vanish as well. In any case, it is obvious that the fixation threshold diverges for $\delta_d \to 0$, as C-agents stop playing with D-neighbors after the first game round, so that neither C's nor D's will be interested in changing strategy afterwards. The bound converges for large h to R^∞_{fix} (from above under condition (1); divide numerator and denominator of (S14) by h and recall that $S^h_{\mathrm{CC}}(1)/h \to 1$ and $S^h_{\mathrm{CD}}(1)/h \to P^\infty_{\mathrm{CD}}$ as $h \to \infty$).

S8 The effect of the predictive horizon h

From eqs. (S7) and (S10), it follows that the contributions of one more prediction step to the expected payoff gains $\Delta\pi^h_{\mathrm{C}}$ and $\Delta\pi^h_{\mathrm{D}}$ computed by agent i are

$$\Delta\pi^{h+1}_{\mathrm{C}} - \Delta\pi^h_{\mathrm{C}} = -r\sum_{p_{ij}=1}\underbrace{\left(P^{h+1}_{\mathrm{CC}}(t_{ji}) - P^{h+1}_{\mathrm{CD}}(t_{ji})\right)}_{>0 \text{ for } h\geq 1} + \sum_{p_{ij}=1} P^{h+1}_{\mathrm{CC}}(t_{ji}) + \sum_{p_{ij}<1} P^{h+1}_{\mathrm{CD}}(t_{ij}) \tag{S15}$$

and

$$\Delta\pi^{h+1}_{\mathrm{D}} - \Delta\pi^h_{\mathrm{D}} = r\sum_{p_{ji}<1}\underbrace{\left(P^{h+1}_{\mathrm{CC}}(t_{ji}) - P^{h+1}_{\mathrm{CD}}(t_{ji})\right)}_{>0 \text{ for } h\geq 1} - \sum_{p_{ji}<1} P^{h+1}_{\mathrm{CC}}(t_{ji}) - \sum_{p_{ji}=1} P^{h+1}_{\mathrm{CD}}(1). \tag{S16}$$

The effect on cooperation is positive, i.e., $\Delta\pi^h_{\mathrm{C}}$ and $\Delta\pi^h_{\mathrm{D}}$ are respectively decreasing and increasing with $h \geq 1$, if

$$r > 1 + \frac{\sum_{p_{ij}=1} P^{h+1}_{\mathrm{CD}}(t_{ji}) + \sum_{p_{ij}<1} P^{h+1}_{\mathrm{CD}}(t_{ij})}{\sum_{p_{ij}=1}\left(P^{h+1}_{\mathrm{CC}}(t_{ji}) - P^{h+1}_{\mathrm{CD}}(t_{ji})\right)} \quad \text{and} \quad r > 1 + \frac{\sum_{p_{ji}<1} P^{h+1}_{\mathrm{CD}}(t_{ji}) + \sum_{p_{ji}=1} P^{h+1}_{\mathrm{CD}}(1)}{\sum_{p_{ji}<1}\left(P^{h+1}_{\mathrm{CC}}(t_{ji}) - P^{h+1}_{\mathrm{CD}}(t_{ji})\right)}, \tag{S17}$$

obtained by solving $\Delta\pi^{h+1}_{\mathrm{C}} - \Delta\pi^h_{\mathrm{C}} < 0$ and $\Delta\pi^{h+1}_{\mathrm{D}} - \Delta\pi^h_{\mathrm{D}} > 0$ for r from eqs. (S15) and (S16).

As discussed in the previous section, the denominators in (S17) are minimized by $t_{ji} = 1$. Since the quantity $P^{h+1}_{\mathrm{CC}}(1) - P^{h+1}_{\mathrm{CD}}(1)$ increases with h (and converges to $1 - P^\infty_{\mathrm{CD}}$ for large h, see Fig. S9**b**), a lower bound to the denominators in (S17)

is $P^2_{\rm CC}(1)-P^2_{\rm CD}(1)=\delta_d(1-\delta_d)$ (see Table S1) to be multiplied by the number of C-neighbors expected by agent i, say $k_{\rm C}$ (the number of p_{ij}'s $=1$ in the left condition; the number of p_{ji}'s <1 in the right one). An upper bound to the numerators is $k(1+3\delta_d)/4$, obtained by replacing $P^{h+1}_{\rm CD}(t_{ji})$, $P^{h+1}_{\rm CD}(t_{ij})$, and $P^{h+1}_{\rm CD}(1)$ with the maximal value assumed by the right-hand side of the recursion in (S2).

We then have $\Delta\pi^{h+1}_{\rm C}-\Delta\pi^h_{\rm C}<0$ and $\Delta\pi^{h+1}_{\rm D}-\Delta\pi^h_{\rm D}>0$ for any $h\geq 1$ under

$$r>1+\frac{k}{k_{\rm C}}\frac{1+3\delta_d}{4\delta_d(1-\delta_d)}. \tag{S18}$$

Condition (S18) is also very conservative for small δ_d. Moreover, even if $\Delta\pi^h_{\rm C}$ (resp. $\Delta\pi^h_{\rm D}$) increases (resp. decreases) with h at small h, it turns decreasing (resp. increasing) for sufficiently large h under condition (1) in the main text. Indeed, the right-hand sides of the conditions in (S17) converge to the right-hand side of (1) for large h.

S9 Two relevant probabilities for strategy update

Two probabilities regarding the process of strategy update have been used in discussing the analytical results in the main text. The first, $P_{1,k}$, is the probability that, in a (possibly infinite) sequence of game rounds, one or more of the $k\geq 1$ neighbors of the focal agent i revise their strategy before i does. The second, $P_{2,k}$, is the probability that, in a (possibly infinite) sequence of game rounds in a star configuration, at least half of the $k\geq 2$ leaves revise their strategy (at least once) before central agent does (k even).

To compute these two probabilities, recall that, after each game round, the probability that an agent revises her strategy is δ, so that the probability that she does not while some of her k neighbors do, is

$$P'_k=(1-\delta)\big(1-(1-\delta)^k\big). \tag{S19}$$

Then, $P_{1,k}$ is obtained as the following geometric series

$$P_{1,k}=\sum_{t=0}^{\infty}(1-\delta)^{t(k+1)}P'_k=P'_k\frac{1}{1-(1-\delta)^{k+1}}=1-\frac{\delta}{1-(1-\delta)^{k+1}}, \tag{S20}$$

i.e., the sum of the probabilities $(1-\delta)^{t(k+1)}$ that no strategy update occurs in the neighborhood up to round $t+1$ of the sequence, each multiplied by the probability P'_k that i does not revise after round $t+1$ while some of the neighbors do. Up to 1-st-order in δ, $P_{1,k}$ behaves as $1-1/(k+1)-(k/2)/(k+1)\delta$, so that it approaches $1-1/(k+1)$ from below for small δ.

The probability $P_{2,k}$ is too complex to be characterized analytically. However, for small δ, we can consider that either none or at most one of the k leaves of the star does revise strategy after each game round, i.e., we neglect the probability $\frac{1}{2}k(k-1)\delta^2(1-\delta)^{k-2}$ of two leaves revising with respect to $k\delta(1-\delta)^{k-1}$ of just one. Then,

$$P_{2,k}\simeq\prod_{k'=k/2+1}^{k}P_{1,k'}=1-\frac{1-(1-\delta)^{k/2}}{1-(1-\delta)^{k+1}}\simeq\frac{1}{2}+\frac{1}{2(k+1)}-\frac{k(k+2)}{8(k+1)}\delta\quad\text{for small }\delta, \tag{S21}$$

i.e., the product of the probabilities $P_{1,k'}$ that one of the k' leaves who still have to revise, does so before the central agent. Thus, $P_{2,k}>0.5$ for small δ, whereas it obviously drops to zero as $\delta\to 1$ (for $\delta=1$, all agents revise their strategies after each game round). Numerically (with a Monte-Carlo approach), we have checked that for $\delta=0.05$ (the reference value used in most of our simulations, see Table 1) $P_{2,k}$ remains larger than 0.5 for stars with up to 70 nodes, that is not far from the maximal degree of the scale-free networks used in our simulations (see Table S2).

S10 Stalemate and fluctuations in the network of Fig. 1b

Consider the network and the initial state of Fig. 1**b**, with $i=1$, $j=2$, nodes 3 to $k_1{+}1$ being the $k_1{-}1$ initial C-neighbors of 1, and nodes $k_1{+}2$ to $k_1{+}k_2$ being the k_2-1 initial D-neighbors of 2, $k_1,k_2>2$.

After the first game round, at which all agents play the PD (i.e., no C abstains from playing), if the C-agent 1 revises her strategy, she remains C if

$$r>r^0_{\rm C,1}=1+\frac{(k_1{-}1)S^h_{\rm CD}(0)+S^h_{\rm CD}(1)}{(k_1{-}1)(h-S^h_{\rm CD}(0))}=1+\frac{S^h_{\rm CD}(0)}{h-S^h_{\rm CD}(0)}+\frac{1}{k_1{-}1}\frac{S^h_{\rm CD}(1)}{h-S^h_{\rm CD}(0)} \tag{S22}$$

(from inequality (S8), taking into account that 1 lowered to p_1 the probability to play with 2 at second round, i.e., $t_{12}=1$). The revising D-agent 2 changes to C if

$$r>r^0_{\rm D,2}=1+k_2\frac{S^h_{\rm CD}(1)}{S^h_{\rm CC}(1)-S^h_{\rm CD}(1)} \tag{S23}$$

(from inequality (S11)). Revising C-agents 3 to k_1+1 remain C under

$$r > r^0_{\mathrm{C},3} = 1 + \frac{S^h_{\mathrm{CD}}(0)}{h - S^h_{\mathrm{CD}}(0)}, \tag{S24}$$

(from inequality (S8) with $t_{i1} = t_{1i} = 0$, $i = 3, \dots, k_1+1$), a condition implied by (S22). Revising D-agents k_1+2 to k_1+k_2 remain D because have no C-neighbors.

Assume that no one changes. After the second game round, if 1 played with 2, the situation is the same as after the first round; otherwise, after a consecutive abstentions, the revising C-agent 1 remains C if

$$r > r^a_{\mathrm{C},1} = 1 + \frac{(k_1-1)S^h_{\mathrm{CD}}(0) + S^h_{\mathrm{CD}}(a+1)}{(k_1-1)(h - S^h_{\mathrm{CD}}(0))} = 1 + \frac{S^h_{\mathrm{CD}}(0)}{h - S^h_{\mathrm{CD}}(0)} + \frac{1}{k_1-1}\frac{S^h_{\mathrm{CD}}(a+1)}{h - S^h_{\mathrm{CD}}(0)}, \tag{S25}$$

while the revising D-agent 2 changes to C if

$$r > r^a_{\mathrm{D},2} = 1 + \frac{S^h_{\mathrm{CD}}(a+1) + (k_2-1)S^h_{\mathrm{CD}}(1)}{S^h_{\mathrm{CC}}(a+1) - S^h_{\mathrm{CD}}(a+1)}. \tag{S26}$$

Again, revising C-agents 3 to k_1+1 remain C under (S24) and revising D-agents k_1+2 to k_1+k_2 do not change strategy.

The threshold $r^a_{\mathrm{C},1}$ is larger than $r^0_{\mathrm{C},1}$ for small a ($S^h_{\mathrm{CD}}(a+1)$ grows with $a \geq 0$ as long a is sufficiently small, see Fig. S10) and decreases for increasing k_1. The threshold $r^a_{\mathrm{D},2}$ decreases as a increases with sufficiently large k_2 ($S^h_{\mathrm{CC}}(a+1) - S^h_{\mathrm{CD}}(a+1)$ grows with $a \geq 0$ as discussed in Sect. S7) and increases for increasing k_2. For sufficiently large k_1 and k_2, we therefore have $\max_a r^a_{\mathrm{C},1} < \min_a r^a_{\mathrm{D},2}$. However, the opposite relation is also possible for any a, k_1, k_2, provided δ_d is sufficiently small.

If $\max_a r^a_{\mathrm{C},1} < r < \min_a r^a_{\mathrm{D},2}$, the network is in a stalemate. The network can also reach a stalemate. Imagine node 2 to be initially a C, with its condition to remain such after the first round unsatisfied, i.e.,

$$r < 1 + \frac{S^h_{\mathrm{CD}}(0) + (k_2-1)S^h_{\mathrm{CD}}(1)}{h - S^h_{\mathrm{CD}}(0)}, \tag{S27}$$

(possible for sufficiently large k_2) and the condition for 1 to remain C met, that is $r > r^0_{\mathrm{C},3}$ from (S24). Then, if 2 changes to D the network goes in the stalemate.

But the same network can also produce long-term fluctuations. Consider the initial state of Fig. 1**b** with $r^0_{\mathrm{C},1} < r < r^a_{\mathrm{C},1} < \min_a r^a_{\mathrm{D},2}$ for some $a > 0$. Then, the C-agent 1 is the only willing to change after a consecutive abstentions. Once 1 switches to D, she will remain such for some game rounds. If in the meantime the C-neighbors 3 to k_1+1 do not change strategy and abstain for a rounds, the condition for 1 to go back C becomes

$$r > 1 + \frac{(k_1-1)S^h_{\mathrm{CD}}(a+1) + S^h_{\mathrm{CD}}(1)}{(k_1-1)(S^h_{\mathrm{CC}}(a+1) - S^h_{\mathrm{CD}}(a+1))}, \tag{S28}$$

that is satisfied for large enough a ($S^h_{\mathrm{CD}}(a+1)$ converges to $S^h_{\mathrm{CD}}(0)$ for large a, so that the right-hand side of (S28) approaches $r^0_{\mathrm{C},1}$). Once 1 switches back to C after a sufficiently long abstention of nodes 3 to k_1+1, the situation is essentially the one just after the first game round.

The above example shows that long-term fluctuations are possible, though sometime difficult to observe. They might require specific sequences of events. Essentially, once an agent changes strategy and plays a game round, she is not in the condition to switch back. A D changes to C when the probabilities that most of her C-neighbors play in the next rounds are sufficiently high, otherwise she will mostly play with D-neighbors; and the strategy change further raises such probabilities, making the switch to D unattractive in the near future. As well, switching to D lowers the probabilities that the C-neighbors play in the next rounds, thus preventing the near switch to C.

S11 Networks

We used six standard types of networks—three regular and three random—of $N = 1000$ nodes and $M = N\langle k\rangle/2$ links, $\langle k\rangle$ denoting the average degree. For each random type, we generated 100 networks. Details on the networks' structure and generation algorithms are given below. Table S2 reports several structural indicators (averaged over the 100 generated networks for random types). See Ref. 79 for further details on generation and analysis of complex networks.

S11.1 Regular networks

Planar lattices: rectangular lattices of N nodes with degree $k = 4$ (horizontal and vertical links—square lattices) and $k = 8$ (also including diagonal links) with periodic boundary conditions. We used lattices of 40×25 nodes.

Ring lattices: loops of N nodes each connected to the $k/2$ nearest nodes in both left and right directions in the loop. We used only $k = 4$ (the degree k must be an even integer).

Complete network: each node is connected to all $N - 1$ others.

Network	$\langle k\rangle$	σ_k	$k_{\min}$	$k_{\max}$	Diameter	Average distance	Transitivity
Planar lattice	4	0	4	4	32	16.3	0
	8	0	8	8	20	11.3	0.42
Ring lattice	4	0	4	4	250	125.38	0.5
Complete network	999	0	999	999	1	1	1
Watts-Strogatz	4	1.40	2	9.85	9.0	5.32	0.003
	8	1.99	4	15.87	5.3	3.59	0.007
Barabási-Albert	4	5.24	2	82.27	7.32	4.07	0.027
	8	8.81	4	108.6	5.0	3.17	0.037
Holme-Kim	4	5.40	2	89.13	11.06	4.88	0.738
	8	8.96	4	115.8	5.44	3.26	0.284

Table S2. Networks' structural indicators (averaged over 100 networks for random models). Columns $\langle k\rangle$, σ_k, $k_{\min}$, and $k_{\max}$ respectively report the average, standard deviation, min and max of the nodes' degree. Network size $N = 1000$ nodes; number of links $M = N\langle k\rangle/2$.

S11.2 Random networks

Watts-Strogatz (WS) with full rewiring: WS rewiring of all left links of a degree-k ring lattice.[80] We use this model to generate single-scale random networks, i.e., networks with sufficiently narrow degree distribution—small variance—so that the average degree $\langle k\rangle = k$ well describes the 'scale' of the connections. The standard single-scale model is the Erdös–Rényi[79] (ER) random network, where each of the $M = N\langle k\rangle/2$ links is included with probability $p = M/(N(N-1)/2) = \langle k\rangle/(N-1)$ and the binomial degree distribution—binomial$(k, N-1, p)$—converges for large N to the Poisson with parameter $\langle k\rangle$. The resulting network is however disconnected if $\langle k\rangle/N$ is too small (it is disconnected with probability 1 if $p < \ln N/N$, a condition that is met in our simulations with $N = 1000$ and $\langle k\rangle = 4$). Though connectivity can be easily forced, the effects on the degree distribution are not easily quantifiable. We therefore opted for the WS model that, in our setting ($N = 1000$), we have estimated to produce connected networks in more than 99.99% of the cases. Note that the WS model is typically used with low rewiring probability (of the left links) to show the 'small-world' property (significant network transitivity and small diameter), whereas we use it here with full rewiring to better approximate an ER network. Indeed, the resulting degree distribution[81]

$$P(k) = \begin{cases} 0 \quad \text{if } k < \langle k\rangle/2, \text{ otherwise} \\ \text{binomial}(k - \langle k\rangle/2, (N-1)\langle k\rangle/2, 1/(N-1)) \end{cases} \xrightarrow[N\to\infty]{} \begin{cases} 0 \quad \text{if } k < \langle k\rangle/2, \text{ otherwise} \\ (\langle k\rangle/2)^{k-\langle k\rangle/2}/(k-\langle k\rangle/2)!\exp(-\langle k\rangle/2) \end{cases} \tag{S29}$$

is Poissonian-like for large N (see, e.g., Fig. 1**b**).

Barabási-Albert (BA): BA degree-rank preferential attachment of $\langle k\rangle/2$ links.[82] We use this model to generate scale-free random networks, i.e., networks with broad degree distribution—large variance—showing low- and high-connected nodes, in spite of their average degree. The BA algorithm produces networks with degree distribution that is zero for $k < \langle k\rangle/2$ and converges, for large k and N, to a power law with exponent -3, hence showing an large variance (infinite variance in the limit $N \to \infty$). The transitivity—the average over the network's nodes of the fraction of connected neighbor pairs—increases with $\langle k\rangle$ but vanishes as $(\ln N)^2/N$ for large N.[83]

Holme-Kim (HK): HK scale-free networks with tunable transitivity.[84] We use this model to generate scale-free random networks with non-vanishing transitivity for large size. With a tunable probability (that we set to 1), the HK algorithm alternates steps of degree-rank preferential attachment with steps in which a triangle is closed between the new node, the last preferred node and a neighbor of the latter. The resulting transitivity does not vanish with N and decreases as $\langle k\rangle$ increases, because the higher number of triangles closed with larger $\langle k\rangle$ does not compensate for the increased number of possibilities. The theoretical degree distribution for large N is the same of the BA model. However, with respect to a finite BA network, the HK algorithm raises the number of low- and high-connected nodes to the detriment of nodes with intermediate degree (checked on average in our 100 networks). Imagine, e.g., to raise transitivity by rewiring. One option is to increase the number of hub-hub connections, to close triangles with common leaves. This is achieved by detaching the termination of a hub's link to reconnect it to another hub. The latter node gains a link, while the looser moves left in the degree distribution. For another option to increase transitivity in scale-free networks—by introducing the small-world property—see Ref. 83.

S12 Numerical simulations

For each panel of Figs. 2 and S1–S5 and for each value of the predictive horizon ($h = 2, \ldots, 5$), we have first run simulations to identify the thresholds r_{inv} and r_{fix} (all simulations reach all-D for $r \le r_{\text{inv}}$; all-C for $r \ge r_{\text{fix}}$). We have then run simulations for r in the open interval $(r_{\text{inv}}, r_{\text{fix}})$ using an equally spaced grid with resolution of about 0.2 (except for Fig. S2**e** in which the scale of r is larger). For a given network of N nodes (labeled 1 to N) and assigned model parameters (r, δ, h, d) (see Table 1 in the main text), we used the following simulation procedure (implemented in Matlab).

Initial state

Initial C-level: we used either 1% or 50%. Let N_C denote the number of initial C's, i.e., $N_C = 10$ or 500 for our networks of $N = 1000$ nodes.

Random placement of the initial C's: The first N_C nodes of a random (uniform distribution) permutation of the integers $\{1,\ldots,N\}$ are set to C; all others are set to D.

Degree-rank placement of the initial C's: the N_C nodes with highest degree are set to C (random choice, if needed, among the nodes sharing the smallest selected degree); all others are set to D.

Probabilities to play: $p_{ij} = p_{ji} = 1$ for all connected pairs (i, j); $p_{ij} = p_{ji} = 0$ otherwise.

Random number generation

For each simulation, we used two independent random number generators, one to generate the network (for random network models) and the initial state, and one to perform the simulation (game rounds and strategy update). The pair of initialization seeds for the two generators uniquely identify the simulation and are stored to allow reproduction.

Game round

At each game round, the interaction between the pair (i, j) of neighbors occurs as follows:

1. if $p_{ij} = 1$, agent i is ready to play the PD; otherwise a random number p_i in $[0,1]$ is drawn (uniform distribution) and i agrees to play the PD with j if $p_i \le p_{ij}$;
2. if $p_{ji} = 1$, agent j is ready to play the PD; otherwise a random number p_j in $[0,1]$ is drawn (uniform distribution) and j agrees to play the PD with i if $p_j \le p_{ji}$;
3. if both i and j agree to play the PD, they play C or D accordingly to their current strategy; from the obtained payoff they get to know the opponent's strategy;
4. if both i and j play C, they respectively set $p_{ij} = 1$ and $p_{ji} = 1$; if i is exploited by j, she sets $p_{ij} = \delta_d$; if j is exploited by i, she sets $p_{ji} = \delta_d$;
5. if i (resp. j) abstains from playing the PD (recall that i and j cannot both abstain, because either $p_{ij} = 1$ or $p_{ji} = 1$ by construction), i (resp. j) updates p_{ij} (resp. p_{ji}) with the recursion (S1); i (resp. j) gets no information on the strategy of j (resp. i), while j (resp. i) gets to know that i (resp. j) is a C. This is simply implemented by assuming that j (resp. i) knows the probability p_{ij} (resp. p_{ji});

Strategy update

After each game round, the strategy update for agent i occurs as follows:

1. a random number p_i in $[0,1]$ is drawn (uniform distribution); if $p_i \le \delta$, then agent i revises her strategy by following the procedure in Sect. S4 if i is a C; the procedure in Sect. S5 if i is a D;
2. if i changes from C to D, she sets $p_{ij} = 1$ toward each neighbor j; if i changes from D to C, she sets $p_{ij} = \delta_d$ toward each neighbor j with $p_{ji} = 1$.

Simulation length and outcome

Max. number of game rounds: $500/\delta$ game rounds ($= 10^4$ for our reference value of δ). It is the time length within which each agent revises strategy 500 times, on average.

Early termination: termination in all-C or all-D before the last game round. There is no reason to continue the simulation, as all-C and all-D are both invariant states (trivial stalemates).

Outcome: 1/0 in case of termination in all-C/D; otherwise, the average C-level (fraction of C-nodes) over the last $100/\delta$ (20% of) game rounds (after strategy update).

Classification

Trivial stalemate: termination in all-C or all-D.

Nontrivial stalemate: termination different from all-C and all-D with no strategy change in the last $100/\delta$ (20% of) game rounds. There is of course no guarantee that this criterion identifies real stalemates. In practice, we classify as nontrivial stalemates the cases in which evolution slows down so much to be in a 'practical' stalemate.

Long-term fluctuation: termination different from all-C and all-D with some strategy change in the last $100/\delta$ game rounds yielding a regression slope within 10^{-6} (the slope of the regression line over the last $100/\delta$ game rounds does not exceed ± 1 agent over 1000 game rounds). This is also a practical criterion.

Non-convergence: termination different from all-C and all-D with some strategy change in the last $100/\delta$ game rounds yielding a regression slope exceeding $\pm 10^{-6}$.

Results

We applied the above classifications to all simulations of Figs. 2 and S1–S5 performed for $r_{\text{inv}} < r < r_{\text{fix}}$, i.e., for values of the game return r and the predictive horizon h for which neither all simulations ended in all-C nor in all-D. The results, grouped by type of network and initialization (i.e., by figure panel) are reported in Table S3. (With respect to the predictive horizon h within each panel, we note that the occurrence of stalemates and fluctuations and the corresponding levels of C slightly increase up to $h = 4$ or 5). Only for Fig. 2, the simulations not ended in all-C or all-D have been extended to a ten-times longer timescale to validate the classification (see rows labeled 'l.t.' in Table S3).

Trivial stalemates

- They constitute a significant fraction of outcomes independently of the network's structure and initialization, all-D dominating close to r_{inv}, all-C close to r_{fix}.
- They are majority starting from 1% initial C's, especially in regular and single-scale networks, and their frequencies slightly increase (especially all-C) by extending the simulations on a longer timescale (compare regular with 'l.t.' rows in the classification of Fig. 2), not only because of the reduced fractions of non-convergent simulations, but also because apparent nontrivial stalemates and fluctuations eventually end up in all-C or all-D.
- They seem to occur less frequently for 50% initial C's (see the classification of Figs. S1 and S2**c**,**d**,**f**), though part of the effect is due to the slower convergence (see the fractions of non-convergent simulations and the comment below on convergence).
- They are more frequent at higher connectivity (compare $\langle k \rangle = 4$ with $\langle k \rangle = 8$) to the detriment of nontrivial stalemates and fluctuations, but also because of the faster convergence.
- Increasing network heterogeneity (from single-scale to scale-free networks) gives more room to nontrivial outcomes. Moreover, all-C relatively gains over all-D with random placement of the initial C, whereas the opposite seems to occur for degree-rank-C-placement. This is perhaps due to the higher/lower r-values in the first/second case. At higher/lower game returns, D's require more/less C-neighbors to change strategy (see condition (1) in the main text), so that convergence to all-D from 1% initial C's is less/more likely. Moreover, the evolution of cooperations is faster/slower (see below the comment below on convergence), and the fraction of non-convergent simulations is accordingly smaller/larger.

Nontrivial stalemates

- They are infrequent and occur at low levels of C starting from 1% initial C's, especially in regular and single-scale networks, with the exception of the complete network. And they are even less frequent if the classification is performed on a longer timescale (compare regular with 'l.t.' rows in Fig. 2).
- They occur at medium-high C-levels starting from 50% initial C's, and they seem to be particularly frequent in high-connected lattices (Fig. S1**b**) and in low-connected scale-free networks with degree-rank-C-placement (Fig. S1**i**).
- In general, while stalemates at low C-levels require high game returns (to allow C's with few C-neighbors to remain C's), stalemates at high C-levels require low returns (not allowing the invasion of cooperation, otherwise invasion reduces the requirement on r for the further spread of C's and evolution cannot halt in a stalemate). The latter are hence reachable only from significant initial C-levels.
- In the complete network, stalemates occur at the initial state (see the mean and variance of the C-level for Fig. S2**e**,**f**). Indeed C's and D's all behave in the same condition and the gap between the r-values above which C's do not change and D's do change is common to all agents.
- The same gap is possible in high-connected lattices for game returns not allowing invasion.
- In scale-free networks, low returns allow a few hubs to remain defectors and the effect is even more evident under degree-rank-C-placement (see the fraction and mean level of stalemates for Fig. S1**i**), because of the lower value of r.
- HK scale-free networks more easily fall in stalemate with respect to BA ones, at higher C-levels, essentially because HK-hubs share more common leaves and this better supports stalemates in which some D-hubs exploit leaves that nonetheless remain C's.
- Nontrivial stalemates are definitely less frequent at higher connectivity (compare $\langle k \rangle = 4$ with $\langle k \rangle = 8$), because of the increased stalemate constraints, but also because the faster convergence (see below the comment below on convergence).
- Contrary to what happens starting from 50% initial C's, degree-rank placement reduces the possibility for nontrivial stalemates from 1% initial C's (in both BA and HK scale-free networks for $\langle k \rangle = 4$, compare Figs. 2**g**,**i** and S5**a**,**c**), as it reduces the game return that should be sufficiently large to allow low stalemates.

Long-term fluctuations

- They are infrequent and occur at low/high C-levels more or less as nontrivial stalemates do.

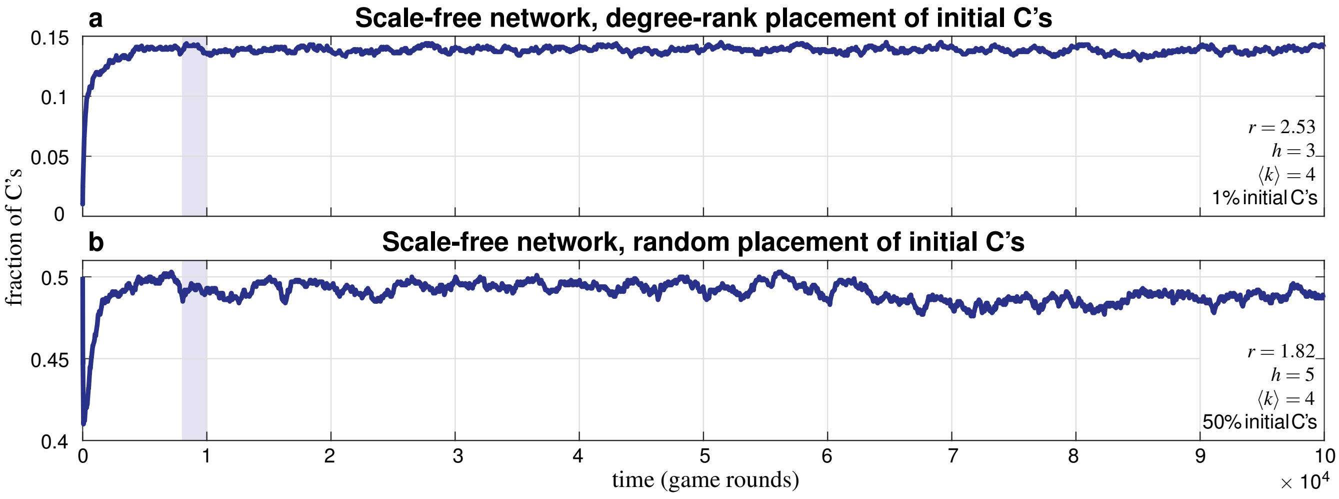

Figure S11. Long-term fluctuations. Two examples observed in scale-free (BA) networks starting from 1% randomly placed (**a**) and 50% degree-rank placed (**b**) initial C's. Other parameters: reference values in Table 1 (main text). Both simulations are classified as long-term fluctuations in the timescale of 10^4 game rounds, i.e., based on rounds from 0.8 to 1 ($\times 10^4$, shaded area); average asymptotic C-level 0.140 (**a**), 0.492 (**b**); fluctuation amplitude 0.008 (**a**), 0.011 (**b**).

- Their amplitude (the min-max excursion of the asymptotic C-level) is always very limited. Most fluctuations are hence practical stalemates of the evolutionary process. Figure S11 shows two relevant examples (with largest amplitude), obtained for 1% (**a**) and 50% (**b**) initial C's.
- Most of the comments reported for nontrivial stalemates apply to long-term fluctuations. In particular, starting from 1% initial C's, stalemates and fluctuations occur at low C-levels. With the exception of HK scale-free networks, the C-level remains below 0.1 on average, with peaks at 0.2, so that the outcomes at medium-high C-levels (dots above 0.2 in Figs. 2, S2**a**, S3, and S4) most likely correspond to non-convergent simulations with increasing regression slope, that should reach all-C on a longer timescale. This indeed happens in most of (the few) cases in Fig. 2 by ten-folding the timescale.
- Note that the confounded effect of the type of placement is faded if the fractions of nontrivial stalemates and fluctuations are considered together.

Non-convergent simulations

- Starting from 1% initial C's, the fractions of non-convergent simulations (columns 'n.c.+' and 'n.c.−' in Table S3) are sufficiently small to justify our timescale of 500 strategy updates, on average, for each agent, with the exception of degree-4 ring lattices (Fig. S2**a**).
- Our primary goal is to identify the invasion and fixation thresholds r_{inv} and r_{fix}, i.e., starting from 1% initial C's. To allow fixation starting from low C-levels, we have therefore used a timescale a few times longer, in terms of number of individual strategy updates, than the network's diameter (see Table S2). The degree-4 ring lattice is indeed the only network requiring the longer timescale. However, looking at the dots at zero C in Fig. S2**a**, we see that all-D outcomes are present up to the identified r_{fix}.
- Non-convergent simulations are more frequent starting from 50% initial C's. The evolution of cooperation is indeed slower, because the values of r for which cooperation persists from 50% are lower than those for which cooperation invades. At low r, a D needs to have a significant fraction of C-neighbors to change to C (see condition (1) in the main text), so she will wait longer (i.e., more strategy updates) before opting for a strategy change, compared to a case with higher r. In contrast, if r allows the invasion of C's, D's are willing to change strategy with few C-neighbors, and this speeds up the spread of C's. The classification of simulations starting from 50% initial C's is therefore imprecise on our timescale. It however gives the interesting insights discussed above on nontrivial stalemates and fluctuations.
- The connectivity of the network speeds up the evolution of cooperation (compare the fractions of non-convergent simulations for $\langle k \rangle = 4$ and $\langle k \rangle = 8$), especially if starting from 1% initial C's. More connections favor the spread of the better strategy and the higher values of r required for the invasion of cooperations further contribute to the faster convergence.
- Network heterogeneity seems to slow down the evolution of cooperation, because of possible bottlenecks in the network structure. The effect is amplified under degree-rank-C-placement, as the thresholds r_{inv} and r_{fix} are lower with respect to single-scale networks.

- Finally, note that the majority of non-convergent simulations have positive regression slope, meaning that the C-level observed on our timescale is underestimated. This is confirmed by the extended simulations performed for Fig. 2 (compare regular with 'l.t.' rows in Table S3), where all-C systematically gains over the other outcomes.

Fig.	Network	Placement of initial C's	Case	Classification (%)					
				all-C	all-D	stalemate [$\langle \mathrm{C} \rangle$, σ_C]	fluctuation [$\langle \mathrm{C} \rangle$,$\sigma_\mathrm{C}$,$\langle \mathrm{A} \rangle$,$\sigma_\mathrm{A}$]	n.c.+	n.c.−
2**a**	planar lattice	random 1%	$\langle k \rangle = 4$	47.50	51.67	0.39 [.004, 0]	0 [–, –, –, –]	0.44	0
l.t.				47.94	51.67	0.28 [.004, 0]	0.11 [.004, 0, .002, 0]	0	0
2**b**			$\langle k \rangle = 8$	44.56	55.44	0 [–, –]	0 [–, –, –, –]	0	0
l.t.				44.56	55.44	0 [–, –]	0 [–, –, –, –]	0	0
2**c**	single-scale	random	$\langle k \rangle = 4$	49.76	36.06	6.24 [.005, .003]	4.53 [.010, .007, .001, .001]	3.35	0.06
l.t.				56.01	36.82	2.29 [.004, .003]	4.53 [.010, .008, .003, .004]	0.35	0
2**d**			$\langle k \rangle = 8$	55.41	42.97	0.31 [.003, .001]	1.11 [.006, .004, .002, .001]	0.17	0.03
l.t.				56.28	43.21	0 [–, –]	0.48 [.004, .002, .002, .002]	0.03	0
2**e**	single-scale	degree-rank	$\langle k \rangle = 4$	60.59	30.23	3.05 [.007, .006]	3.95 [.012, .010, .001, .001]	2.18	0
l.t.				64.18	30.95	0.59 [.004, .002]	4.01 [.016, .015, .004, .005]	0.27	0
2**f**			$\langle k \rangle = 8$	60.63	38.20	0.03 [.004, 0]	0.97 [.005, .003, .002, .001]	0.17	0
l.t.				61.33	38.37	0 [–, –]	0.30 [.007, .003, .002, .001]	0	0
2**g**	scale-free	random	$\langle k \rangle = 4$	57.30	22.38	11.75 [.013, .013]	4.78 [.019, .017, .002, .001]	3.76	0.03
l.t.				63.00	22.81	7.30 [.013, .014]	6.35 [.024, .023, .002, .003]	0.54	0
2**h**			$\langle k \rangle = 8$	63.70	29.53	1.37 [.005, .005]	3.84 [.010, .006, .002, .001]	1.43	0.13
l.t.				67.23	30.03	0.54 [.004, .006]	2.13 [.012, .007, .004, .004]	0.07	0
2**i**	scale-free	degree-rank	$\langle k \rangle = 4$	30.31	37.00	5.54 [.014, .014]	14.62 [.064, .046, .003, .002]	10.76	1.77
l.t.				38.69	37.38	4.08 [.012, .014]	18.70 [.070, .047, .006, .004]	1.15	0
2**j**			$\langle k \rangle = 8$	56.24	35.06	2.00 [.005, .002]	4.06 [.013, .014, .002, .001]	2.35	0.29
l.t.				59.48	36.35	0.29 [.006, .001]	3.76 [.016, .014, .005, .004]	0.12	0
S1**a**	planar lattice	random 50%	$\langle k \rangle = 4$	12.25	12.50	3.25 [.271, .056]	10.13 [.300, .070, .002, .001]	38.37	23.50
S1**b**			$\langle k \rangle = 8$	16.58	18.67	24.33 [.098, .090]	3.50 [.246, .074, .002, .001]	26.50	10.42
S1**c**	single-scale	random	$\langle k \rangle = 4$	13.00	35.40	2.80 [.099, .148]	14.30 [.333, .085, .003, .002]	20.70	13.80
S1**d**			$\langle k \rangle = 8$	0	52.00	1.00 [.351, 0]	19.50 [.377, .035, .002, .001]	7.00	20.50
S1**e**	single-scale	degree-rank	$\langle k \rangle = 4$	64.05	5.72	4.67 [.756, .174]	5.06 [.570, .176, .003, .002]	12.50	8.00
S1**f**			$\langle k \rangle = 8$	11.80	23.00	0.40 [.483, .127]	13.80 [.516, .044, .002, .001]	45.40	5.60
S1**g**	scale-free	random	$\langle k \rangle = 4$	29.74	22.67	1.17 [.311, .280]	6.42 [.500, .118, .004, .003]	35.17	4.83
S1**h**			$\langle k \rangle = 8$	39.88	17.67	0.56 [.427, .087]	5.89 [.397, .072, .002, .001]	33.11	2.89
S1**i**	scale-free	degree-rank	$\langle k \rangle = 4$	19.60	10.20	40.10 [.861, .106]	21.30 [.940, .057, .002, .001]	7.00	1.80
S1**j**			$\langle k \rangle = 8$	0.83	18.17	0.67 [.649, .126]	2.33 [.719, .216, .002, .001]	65.17	12.83
S2**a**	ring lattice	random 1%	$\langle k \rangle = 4$	26.76	51.12	0 [–, –]	0 [–, –, –, –]	22.12	0
S2**b**			$\langle k \rangle = 8$	41.72	58.19	0 [–, –]	0 [–, –, –, –]	0.09	0
S2**c**	ring lattice	random 50%	$\langle k \rangle = 4$	10.50	8.83	9.34 [.270, .096]	10.33 [.269, .123, .001, .001]	55.83	5.17
S2**d**			$\langle k \rangle = 8$	0.75	0	41.38 [.230, .099]	10.12 [.369, .212, .002, .001]	43.50	4.25
S2**e**	complete	random 1%		60.80	26.80	11.20 [.010, 0]	1.20 [.010, .000, .002, .001]	0	0
S2**f**		random 50%		0	0	99.60 [.500, .000]	0.40 [.501, .001, .001 0]	0	0
S3**a**	planar lattice	random 1%	$d = \{-1, 0, .5\}$	51.43	48.04	0.18 [.004 0]	0 [–, –, –, –]	0.35	0
S3**b**			$\delta = \{.025, .05, .1\}$	47.57	51.05	0.24 [.006 .003]	0 [–, –, –, –]	1.14	0
S3**c**	single-scale	random	$d = \{-1, 0, .5\}$	64.96	27.12	3.32 [.004, .003]	2.80 [.009, .006, .001, .001]	1.80	0
S3**d**			$\delta = \{.025, .05, .1\}$	58.35	31.84	2.81 [.004, .002]	4.15 [.010, .008, .002, .001]	2.85	0
S3**e**	single-scale	degree-rank	$d = \{-1, 0, .5\}$	60.16	29.74	2.63 [.005, .005]	3.68 [.013, .009, .002, .001]	3.58	0.21
S3**f**			$\delta = \{.025, .05, .1\}$	62.21	27.54	2.79 [.005, .004]	3.46 [.012, .009, .002, .001]	3.92	0.08
S3**g**	scale-free	random	$d = \{-1, 0, .5\}$	46.80	30.09	13.37 [.012, .013]	5.47 [.019, .018, .001, .001]	4.27	0
S3**h**			$\delta = \{.025, .05, .1\}$	49.68	25.36	14.89 [.011, .011]	5.21 [.019, .019, .001, .001]	4.86	0
S3**i**	scale-free	degree-rank	$d = \{-1, 0, .5\}$	32.93	33.36	6.14 [.020, .024]	14.71 [.057, .038, .002, .001]	11.50	1.36
S3**j**			$\delta = \{.025, .05, .1\}$	45.22	27.67	5.61 [.019, .027]	12.06 [.054, .038, .002, .001]	8.72	0.72
S4**a**	planar lattice	random pair 1%	$\langle k \rangle = 4$	20.14	73.64	2.29 [.006, .002]	0.64 [.012, .008, .002, .001]	3.29	0
S4**b**			$\langle k \rangle = 8$	46.69	53.25	0.03 [.004, 0]	0 [–, –, –, –]	0.03	0
S4**c**	single-scale	random pair	$\langle k \rangle = 4$	27.42	47.50	8.83 [.006, .004]	9.92 [.012, .010, .002, .001]	6.04	0.29
S4**d**			$\langle k \rangle = 8$	53.59	42.56	0.49 [.004, .002]	2.48 [.008, .007, .003, .002]	0.81	0.07
S4**g**	scale-free	random pair	$\langle k \rangle = 4$	37.26	27.06	15.55 [.017, .019]	12.24 [.030, .029, .001, .001]	7.24	0.65
S4**h**			$\langle k \rangle = 8$	42.48	38.12	2.08 [.005, .002]	11.60 [.012, .009, .002, .001]	5.32	0.40
S5**g**	HK scale-free	random 1%	$\langle k \rangle = 4$	35.90	13.82	45.71 [.133, .230]	0.85 [.089, .164, .002, .001]	3.64	0.08
S5**h**			$\langle k \rangle = 8$	53.17	42.63	1.14 [.004, .002]	2.40 [.009, .006, .002, .001]	0.66	0
S5**i**	HK scale-free	degree-rank	$\langle k \rangle = 4$	41.23	10.97	34.69 [.524, .375]	4.80 [.270, .235, .002, .002]	8.14	0.17
S5**j**			$\langle k \rangle = 8$	32.04	55.81	3.77 [.005, .003]	5.92 [.013, .017, .002, .001]	2.15	0.31

Table S3. Classifications of the simulations performed for $r_\mathrm{inv} < r < r_\mathrm{fix}$ grouped by panel of Figs. 2 and S1–S5. For each panel, the table reports the fractions of the six possible outcomes (all-C, all-D, stalemate, fluctuation, non-convergence with positive/negative regression slope). To quantify stalemates and fluctuations, the table reports the mean and standard deviation ($\langle \mathrm{C} \rangle$ and σ_C) of the average asymptotic C-level and, only for fluctuations, the mean and standard deviation ($\langle \mathrm{A} \rangle$ and σ_A) of the oscillation's amplitude (the min-max excursion of the asymptotic C-level). To validate the classification, the simulations of Fig. 2 have been extended over a ten-times longer timescale ($5000/\delta = 10^5$ game rounds, the last 20% of which used for the classification; see row label 'l.t.'), showing no significant change.

Additional references

76. Tanimoto, J. & Sagara, H. Relationship between dilemma occurrence and the existence of a weakly dominant strategy in a two-player symmetric game. *BioSystems* **90**, 105–114 (2007).

77. Wang, Z., Kokubo, S., Jusup, M. & Tanimoto, J. Universal scaling for the dilemma strength in evolutionary games. *Physics of life reviews* **14**, 1–30 (2015).

78. Della Rossa, F., Dercole, F. & Vicini, C. Extreme selection unifies evolutionary game dynamics in finite and infinite populations. *Bull. Math. Biol.* **79**, 1070–1099 (2017).

79. Boccaletti, S., Latora, V., Moreno, Y., Chavez, M. & Hwang, D. H. Complex networks: Structure and dynamics. *Phys. Rev.* **424**, 175–308 (2006).

80. Watts, D. J. & Strogatz, S. H. Collective dynamics of 'small-world networks'. *Nature* **393**, 440–442 (1998).

81. Barrat, A. & Weigt, M. On the properties of small-world network models. *Eur. Phys. J. B* **13**, 547–560 (2000).

82. Barabási, A.-L. & Albert, R. Emergence of scaling in random networks. *Science* **286**, 509–512 (1999).

83. Klemm, K. & Eguiluz, V. Growing scale-free networks with small-world behavior. *Phys. Rev. E.* **65**, 057102 (2002).

84. Holme, P. & Kim, B. J. Growing scale-free networks with tunable clustering. *Phys. Rev. E* **65**, 026107 (2002).
